# Telehealth Education in Allied Health Care and Nursing: Web-Based Cross-Sectional Survey of Students’ Perceived Knowledge, Skills, Attitudes, and Experience

**DOI:** 10.2196/51112

**Published:** 2024-03-21

**Authors:** Lena Rettinger, Peter Putz, Lea Aichinger, Susanne Maria Javorszky, Klaus Widhalm, Veronika Ertelt-Bach, Andreas Huber, Sevan Sargis, Lukas Maul, Oliver Radinger, Franz Werner, Sebastian Kuhn

**Affiliations:** 1 Health Assisting Engineering FH Campus Wien University of Applied Sciences Vienna Austria; 2 Institute of Digital Medicine Philipps-University & University Hospital of Giessen and Marburg Marburg Germany; 3 Competence Center INDICATION FH Campus Wien University of Applied Sciences Vienna Austria; 4 Logopedics – Phoniatrics - Audiology FH Campus Wien University of Applied Sciences Vienna Austria; 5 Physiotherapy FH Campus Wien University of Applied Sciences Vienna Austria; 6 Occupational Therapy FH Campus Wien University of Applied Sciences Vienna Austria; 7 Orthoptics FH Campus Wien University of Applied Sciences Vienna Austria; 8 Midwifery FH Campus Wien University of Applied Sciences Vienna Austria; 9 Competence Center Nursing Sciences FH Campus Wien University of Applied Sciences Vienna Austria

**Keywords:** telehealth, health care education, student perspectives, curriculum, interdisciplinary education

## Abstract

**Background:**

The COVID-19 pandemic has highlighted the growing relevance of telehealth in health care. Assessing health care and nursing students’ telehealth competencies is crucial for its successful integration into education and practice.

**Objective:**

We aimed to assess students’ perceived telehealth knowledge, skills, attitudes, and experiences. In addition, we aimed to examine students’ preferences for telehealth content and teaching methods within their curricula.

**Methods:**

We conducted a cross-sectional web-based study in May 2022. A project-specific questionnaire, developed and refined through iterative feedback and face-validity testing, addressed topics such as demographics, personal perceptions, and professional experience with telehealth and solicited input on potential telehealth course content. Statistical analyses were conducted on surveys with at least a 50% completion rate, including descriptive statistics of categorical variables, graphical representation of results, and Kruskal Wallis tests for central tendencies in subgroup analyses.

**Results:**

A total of 261 students from 7 bachelor’s and 4 master’s health care and nursing programs participated in the study. Most students expressed interest in telehealth (180/261, 69% very or rather interested) and recognized its importance in their education (215/261, 82.4% very or rather important). However, most participants reported limited knowledge of telehealth applications concerning their profession (only 7/261, 2.7% stated profound knowledge) and limited active telehealth experience with various telehealth applications (between 18/261, 6.9% and 63/261, 24.1%). Statistically significant differences were found between study programs regarding telehealth interest (*P*=.005), knowledge (*P*<.001), perceived importance in education (*P*<.001), and perceived relevance after the pandemic (*P*=.004). Practical training with devices, software, and apps and telehealth case examples with various patient groups were perceived as most important for integration in future curricula. Most students preferred both interdisciplinary and program-specific courses.

**Conclusions:**

This study emphasizes the need to integrate telehealth into health care education curricula, as students state positive telehealth attitudes but seem to be not adequately prepared for its implementation. To optimally prepare future health professionals for the increasing role of telehealth in practice, the results of this study can be considered when designing telehealth curricula.

## Introduction

### Background

Telehealth has become increasingly important in recent years, particularly considering technological and societal developments. Telehealth is the use of information and communications technologies to deliver health services where there is a physical separation between care providers or recipients over both long and short distances [1]. It has the potential to help overcome barriers to accessing care, particularly in remote or underserved areas [2], and can be particularly beneficial for patients with chronic diseases [3] to improve long-term adherence [4], as well as addressing shortages in the health care workforce [5]. Owing to the COVID-19 pandemic, the integration of telehealth into the services of health care providers was further increased to prevent further infections and to serve patients in isolation [6-10].

However, the growing use has further highlighted the need for telehealth education for health care providers [11-16]. To successfully and sustainably implement telehealth and subsequently reap the benefits, it is necessary to integrate telehealth into the curricula of future health care providers [5]. A lack of knowledge and experience, as well as a lack of appropriate telehealth training, have been identified as major barriers to telehealth implementation among health care providers [17]. Conversely, telehealth education and training can increase the willingness to adopt telehealth, the perceived readiness, and confidence [5,13,18-20].

Providing telehealth services not only requires a basic understanding of telehealth and its applications but also an assortment of competencies spanning from theoretical knowledge to practical skills, closely mirroring the concepts of Miller pyramid of clinical competence [21] or its adapted version, the Miller prism [22]. As they outline, there are different levels of competence, such as knowledge, skills, and attitudes. In terms of telehealth competencies, *knowledge* involves the basic understanding of telehealth, its tools, and its applications. This also includes knowledge on how to ensure privacy and confidentiality [11,23-27]. The second competence level *skills* refers to the know-how. In telehealth, it requires health care professionals to organize and apply their knowledge to conduct physical assessments via telehealth, make perceptive observation-based examinations, and communicate effectively in a nontraditional clinical setting [5,10,11,15,16,19,23,24,26-29]. In the *performance* or *show* level, professionals demonstrate their ability to select, implement, and use appropriate telehealth tools in a simulated or controlled environment. This is where technological skills become crucial [11,23-27]. Finally, at the *action* or *does* level, health care professionals are expected to perform these skills in real-life situations, providing high-quality and safe telehealth services, and effectively incorporating ethical considerations into their practice [23-25]. *Attitude* is considered a vital component, along with knowledge, skills, and performance, that contributes to actual work competency. It refers to the behavioral and emotional aspects that influence how knowledge and skills are applied in practice [30]. *Attitude* can encompass elements such as motivation, ethical considerations, professionalism, and openness to learning, which seem to be important in the telehealth context.

In accordance with the principles of competency-based frameworks, curricula of health care study programs need to be adapted to qualify health care professionals at all levels of competency, increasing the probability that telehealth is effectively implemented in daily practice [11]. Two reviews [26,31] conducted in 2021 highlighted significant shortcomings in the training and curricula in allied health and nursing. They showed that there was a lack of consistency and absence of a systematic approach in integrating telehealth into these curricula [26,31]. Thus, it is crucial to design telehealth curricula with competency-based frameworks in mind to meet the diverse needs of students and ensure they are equipped with the necessary knowledge, performance skills, and attitude to effectively use telehealth technologies in their future health care practices. An increasing number of standards and guidelines are becoming available to guide the development of individual telehealth courses. They focus on various aspects such as administrative [32,33], ethical [32,34], clinical [32], technical [32,35], or soft skills [36]. However, they often do not address the specialized needs of allied health professionals [37]. Therefore, identifying the specific interests and learning needs of students can help educators to plan their teaching methods and provide tailored curricula or courses in individual study programs. This can further help to promote student engagement and motivation, ensure that the education is relevant and meaningful to their future professional practice, and ultimately improve learning outcomes.

### Aim

The primary objective of this study was to assess the perceived telehealth knowledge, skills, attitude, and experience among health care professionals and nursing students to understand students’ current self-assessed telehealth competencies and identify their learning needs. Our secondary objective was to evaluate students’ preferences for telehealth content and teaching methods withing their respective curricula. This dual focus is intended to provide a rounded perspective of the students’ perceived readiness for telehealth practice and to inform effective educational strategies.

## Methods

### Study Design

We conducted an anonymous cross-sectional web-based survey among the total population of selected health care profession students at *FH Campus Wien* (University of Applied Sciences). Reporting followed the Checklist for Reporting Results of Internet E-Surveys (CHERRIES) [38].

### Sample Characteristics

Given the exploratory nature of this study, the sample size was not predetermined but was derived from the number of students enrolled in the targeted health care and nursing programs who were available and consented to participate during the survey period. At the time of the survey’s release, 2273 students in the following selected academic health care professions were actively studying at the “FH Campus Wien” (University of Applied Sciences, Vienna, Austria) and were thus eligible to participate: BSc dietetics (DIE), BSc occupational therapy (OT), BSc health care and nursing (NUR), BSc midwifery (MID), BSc speech and language therapy (SLT), BSc orthoptics (ORT), BSc physiotherapy (PT), MSc health assisting engineering (HAE), MSc advanced nursing counseling (ANC), MSc advanced nursing education (ANE), and MSc advanced nursing practice (ANP). Participants who answered <50% of the questions were excluded.

### Survey Administration

Students of all semesters were contacted directly with an email invitation. The survey was not listed publicly, no advertisement or incentive offers were put in place, and survey participation was voluntary. The survey was created using the web-based platform LimeSurvey (version 5.3.12 [39]), and it was open for participation between May 2 and May 30, 2022. An invitation email was sent on May 2, 2022. As a measure to improve the response rate, a first reminder was sent on May 9, 2022, and a second reminder was sent on May 25, 2022. Data were stored in a password-secured folder to which only selected study team members had access. Cookies were used to prevent users from accessing the survey twice, and IP addresses were not stored. No other measures to identify multiple entries were used. To ensure anonymous participation no registration process was put in place.

### Ethical Considerations

Anonymous surveys currently do not require a formal review by a research ethics committee under Austrian research governance, in which the Declaration of Helsinki defines applicability to research on identifiable human data [40]. Exemption from ethical review has been formally confirmed by the Ethics Committee of the FH Campus Wien University of Applied Sciences (waiver no. W02/24). The survey followed ethical research practices (ie, voluntary participation; reassurance of anonymity, data protection, and confidentiality; advance information on purpose and content; provision of contact details of the research team; and full disclosure of involved organizations). This information was summarized on the first page of the web-based survey. Anonymous electronic consent to voluntary participation was required to begin the survey, but no signatures were obtained. All data processing procedures have been discussed in detail with the data protection officer of *FH Campus Wien* (University of Applied Sciences, Vienna). All data obtained in this survey will be stored for 10 years in compliance with national research legislation and the funding body.

### Data Collection Methods

We used a newly developed, project-specific questionnaire (Multimedia Appendices 1 and 2). Feedback from project members on topics, constructs, and scales was iteratively incorporated into a first complete survey draft. Subsequent face-validity testing for usability and technical functionality was performed by 3 persons, not involved in the project, requiring minor usability and wording revisions. The survey consisted of 5 pages with 20 questions, of which 16 questions were mandatory: 6 demographical questions (including a question on the self-assessed information and communications technology competence to further describe the technology skills of the sample), 5 questions about personal perceptions of telehealth, 1 question on professional experience with telehealth, and 4 questions on potential content for telehealth courses or curriculum. The 4 optional questions were included to facilitate additional input or clarification.

Eligibility criteria were queried at the beginning of the survey: “Do you study at FH Campus Wien?” and “Which study program do you attend?” Respondents who clicked the survey link but were not eligible were taken directly to the end of the survey. Telehealth interest and perceived importance of telehealth in education were rated on a 4-point Likert scale (1=not interested/important, 2=less interested/important, 3=rather interested/important, and 4=very interested/important), and perceived relevance of telehealth after the pandemic was also rated on a 4-point Likert scale (1=for sure not, 2=rather not, 3=probably, and 4=for sure). Telehealth knowledge was rated by selecting 1 of 5 statements (1=I have never heard of telehealth, 2=I know the term but not more about it, 3=I know telehealth in medical services but not so much about it in my own profession, 4=I know some telehealth applications in my own profession, and 5=I know a lot of telehealth applications in my own profession). Experience with telehealth was rated among the options “performed,” “observed,” and “neither nor” for given examples. The perceived relevance of types of telehealth for the profession was assessed with multiple selections of given examples. Participants rated their interest in telehealth content on a 4-point Likert scale (1=for sure, 2=rather yes, 3=rather not, and 4=for sure not) for given examples. The preferred setting for learning about telehealth was assessed using single choice selection. The option “Don’t know” was implemented, where applicable. Items were not randomized and were always presented in the same order to maintain the survey structure.

### Statistical Analysis

Questionnaires with a completion rate of at least 50% were analyzed. Predefined subgroup analysis to compare for study programs, age, gender, and study year was undertaken. Descriptive statistics of categorical variables were reported as absolute and relative frequencies, and ordinal variables were reported with median. Histograms, heat maps, and boxplots were deployed for graphical illustration of the results. Boxplots display the first and third quartiles as a rectangular box, with whiskers extending from the box to indicate the minimum and maximum values, except for outliers. The median is depicted by a horizontal line. Outliers are represented by individual dots, whereas the mean is denoted using an “x” symbol. Stacked bar charts represent the frequencies of positive (right to 0) and negative responses (left to 0) for categorical variables with higher values in the middle. Kruskal Wallis tests were conducted to test for central tendencies in the subgroup analyses. The α value was set at .05, and exact *P* values were reported. The following mergers were made to achieve a minimum of 5 participants in each subgroup for Kruskal Wallis tests: semesters were merged into study years, for example, first and second bachelor’s semesters combined; age groups were assessed in 8 age group categories (<20, 21-25, 26-30,..., and >50 years) but combined for subgroup analysis into 3 generation groups. In the literature, generational affiliations vary among different publications [41,42]. In this study, Generation Z was defined as students aged up to 25 years, Generation Y encompassed students aged between 26 and 40 years, and students aged ≥41 years were grouped into Generation X and baby boomers. Furthermore, pairwise comparisons were conducted. Test statistic H, SE, standardized test statistic, unadjusted *P* values, and Bonferroni-adjusted *P* values were reported. The Bonferroni-adjusted statistical significance was summarized graphically using spider web figures. Pairwise comparisons were not conducted if the alternative hypothesis was rejected by the overall Kruskal Wallis test.

## Results

### Overview

A total of 2273 students of the selected academic health care professions were potentially eligible to participate. The link to the web-based survey was accessed by 281 students, of whom 261 (92.9%) completed the questionnaire (ie, answered at least 50% of the questions) and were therefore included in the analysis, resulting in a completion rate of 93%. Overall, 206 students were attending a bachelor’s degree program and 55 students were attending a master’s degree program (Table 1). The demographic characteristics of the survey participants are presented in Table 2.

**Table 1:** Participation across the selected bachelor’s and master’s programs (N=261).

Programs			Values, n (%)		Response rate (%)
**Bachelor’s programs**			206 (79)		9
	Dietetics	20 (9.7)		36	
	Occupational therapy	24 (11.7)		24	
	Nursing	32 (15.5)		2	
	Midwifery	35 (17)		31	
	Speech and language therapy	25 (12.1)		37	
	Orthoptics	23 (11.2)		51	
	Physiotherapy	47 (22.8)		13	
**Master’s programs**			55 (21)		32
	Advanced nursing counseling	7 (13)		35	
	Advanced nursing education	16 (29)		27	
	Advanced nursing practice	13 (24)		28	
	Health assisting engineering	19 (34)		40	

**Table 2:** Demographic characteristics of survey participants (N=261).

Characteristics		Total, n (%)	Bachelor’s (n=206), n (%)	Master’s (n=55), n (%)
**Generation Z (years)**		157 (60.15)	151 (73.3)	6 (10.9)
	<20	33 (12.64)	33 (16.02)	0 (0)
	21-25	124 (47.51)	118 (57.28)	6 (10.9)
**Generation Y (years)**		82 (31.42)	51 (24.76)	31 (56.36)
	26-30	48 (18.39)	33 (16.02)	15 (27.27)
	31-35	27 (10.34)	14 (6.8)	13 (23.64)
	36-40	7 (2.68)	4 (1.94)	3 (5.45)
**Generation X, baby boomers (years)**		22 (8.43)	4 (1.94)	18 (32.73)
	41-45	13 (4.98)	4 (1.94)	9 (16.36)
	46-50	3 (1.15)	0 (0)	3 (5.45)
	>50	6 (2.3)	0 (0)	6 (10.9)
**Gender**				
	Man	228 (87.36)	184 (89.32)	44 (80)
	Woman	30 (11.49)	19 (9.22)	11 (20)
	Nonbinary	3 (1.15)	3 (1.46)	0 (0)
**Semester**				
	BSc 1-2	103 (39.46)	103 (50)	N/A^a^
	BSc 3-4	61 (23.37)	61 (29.62)	N/A
	BSc 5-6	42 (16.09)	42 (20.39)	N/A
	MSc 1-2	33 (12.64)	N/A	33 (60)
	MSc 3-4	22 (8.43)	N/A	22 (40)
**Self-assessed ICT^b^ competence^c^**				
	1=very good	80 (30.65)	65 (31.55)	15 (27.27)
	2=good	129 (49.43)	101 (49.03)	28 (50.91)
	3=medium	50 (19.16)	38 (18.45)	12 (21.82)
	4=sufficient	1 (0.38)	1 (0.49)	0 (0)
	5=not sufficient	1 (0.38)	1 (0.49)	0 (0)

^a^N/A: not applicable.

^b^ICT: information and communications technology.

^c^Corresponding to the Austrian school grading system.

### Subgroup Differences

**Figure 1:**
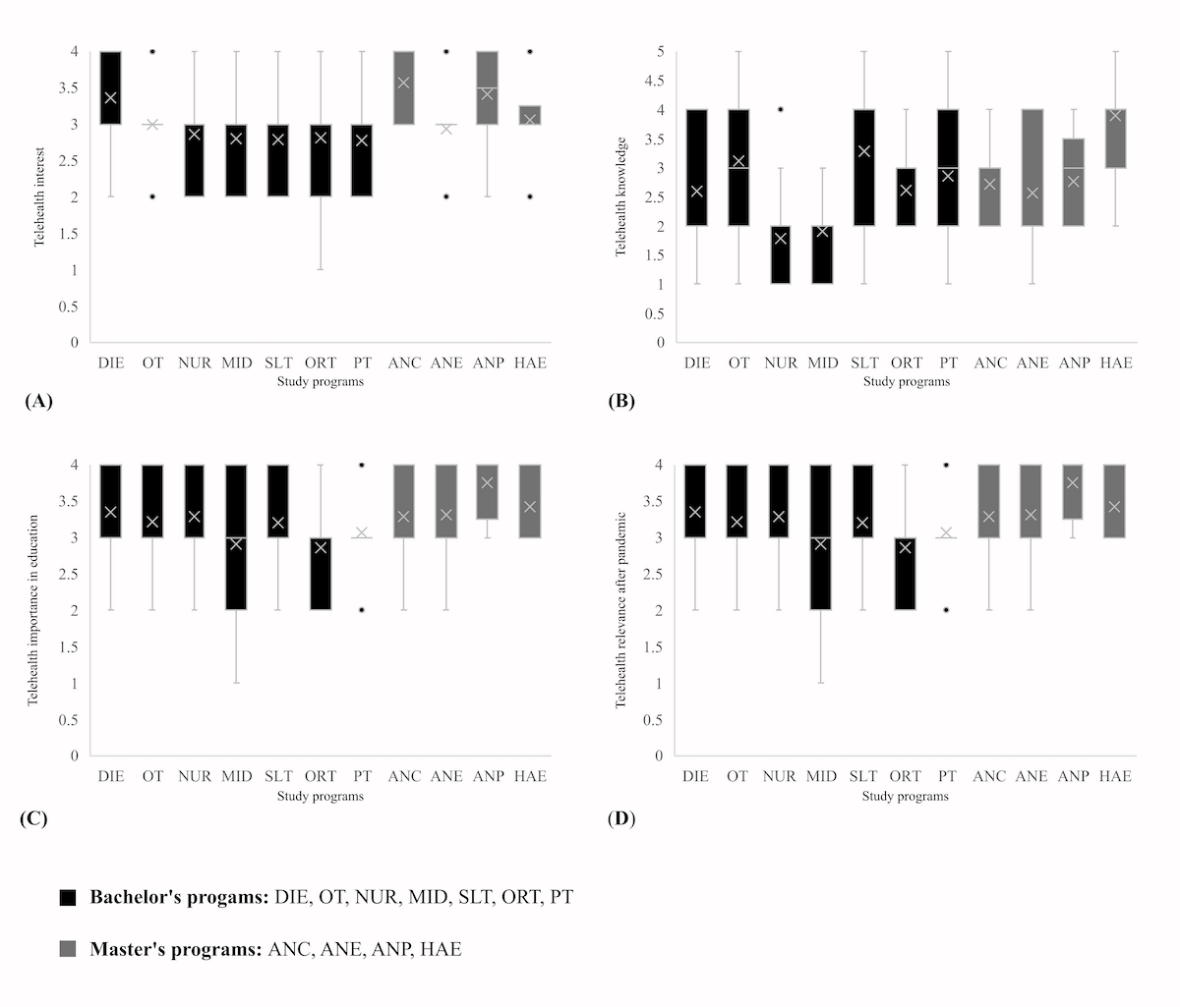
Box plots of (A) telehealth interest, (B) telehealth knowledge, (C) perceived telehealth importance in education, and (D) perceived telehealth relevance after pandemic for each study program. Higher values represent higher interest, knowledge, importance, and perceived relevance. ANC: advanced nursing counseling; ANE: advanced nursing education; ANP: advanced nursing practice; DIE: dietetics; HAE: health assisting engineering; MID: midwifery; NUR: health care and nursing; ORT: orthoptics; OT: occupational therapy; PT: physiotherapy; SLT: speech and language therapy.

**Figure 2:**
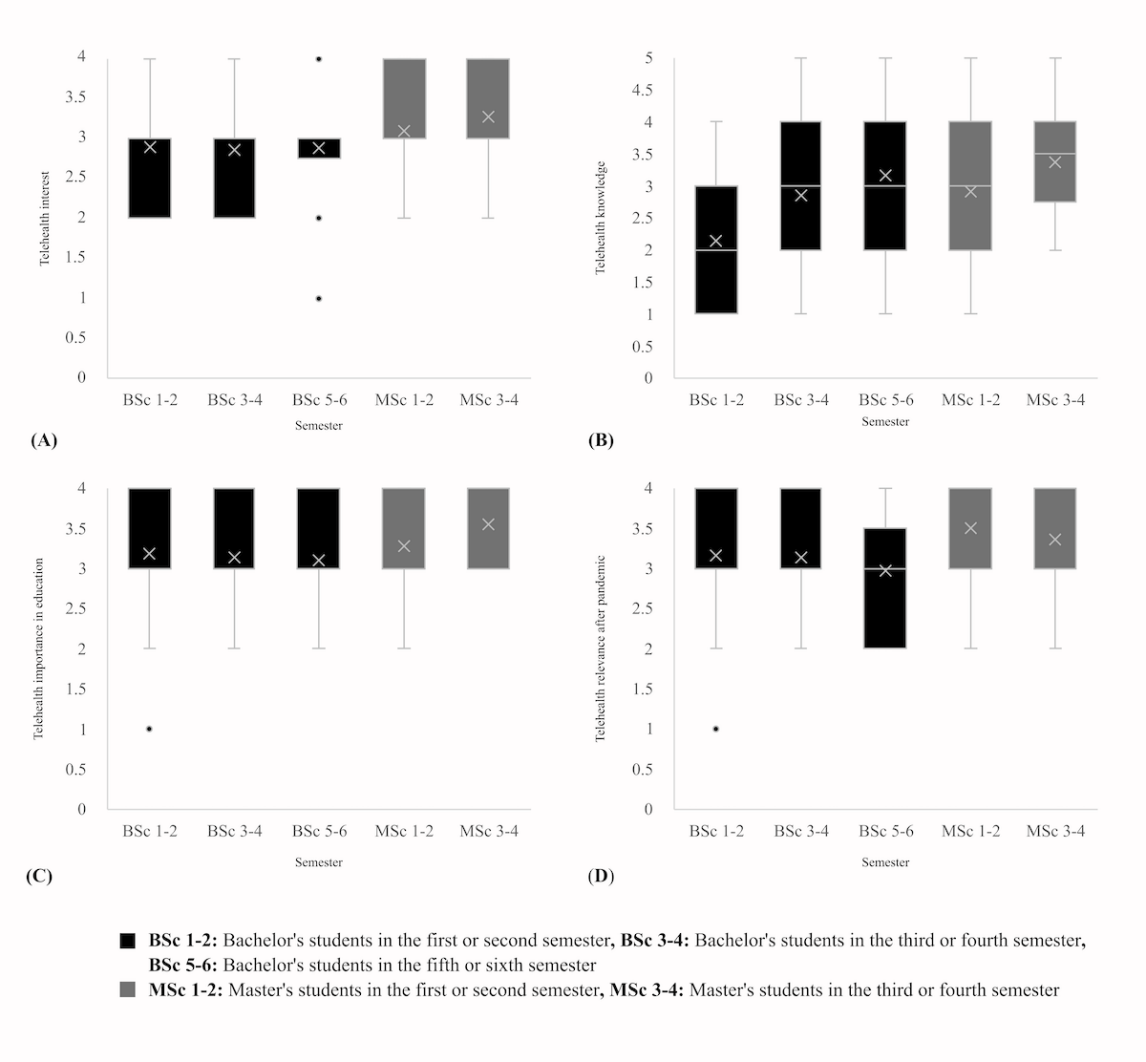
Box plots of (A) telehealth interest, (B) telehealth knowledge, (C) perceived telehealth importance in education, and (D) perceived telehealth relevance after the pandemic, based on semester. Higher values represent higher interest, knowledge, importance, and perceived relevance.

**Figure 3:**
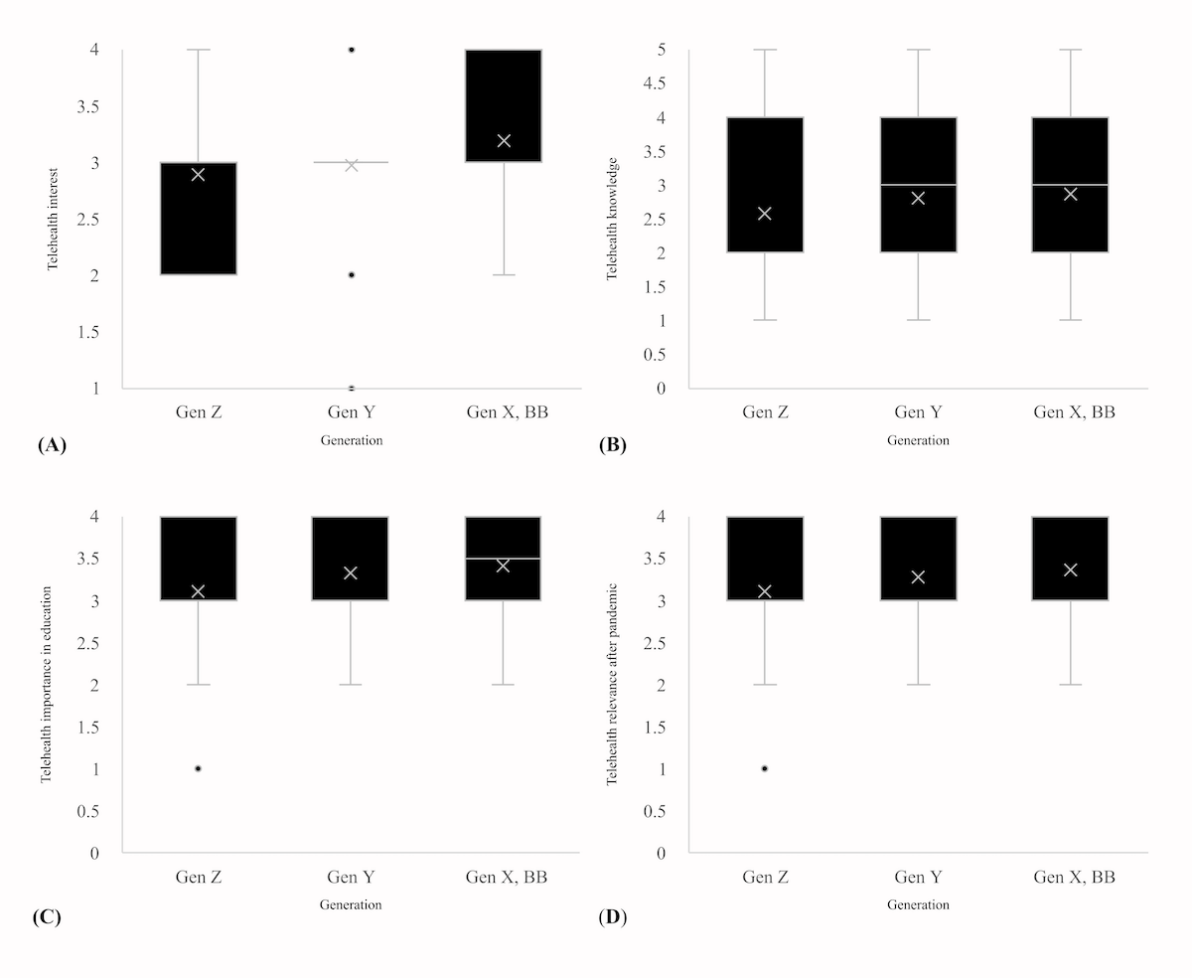
Box plots of (A) telehealth interest, (B) telehealth knowledge, (C) perceived telehealth importance in education, and (D) perceived telehealth relevance after the pandemic for different generations. Higher values represent higher interest, knowledge, importance, and perceived relevance. Gen Z (Generation Z): up to 25 years; Gen Y (Generation Y): 26-40 years; Gen X, BB (Generation X, baby boomer): ≥41 years.

A Kruskal Wallis H test (Table 3) showed that there was a statistically significant difference between the study programs in telehealth interest (*P*=.005), telehealth knowledge (*P*<.001), perceived importance of telehealth in education (*P*<.001), and perceived relevance of telehealth after the pandemic (*P*=.004). Corresponding box plots are shown in Figures 1-3. There were no significant differences between genders in telehealth interest (*P*=.63), telehealth knowledge (*P*=.19), perceived importance of telehealth in education (*P*=.73), and perceived relevance of telehealth after the pandemic (*P*=.55). On the basis of age and generation, there were significant differences in the perceived importance of telehealth education (*P*=.01) but no significant differences in telehealth interest (*P*=.14), telehealth knowledge (*P*=.19), and perceived relevance of telehealth after the pandemic (*P*=.06). There was a significant difference between students of different semesters in telehealth knowledge (*P*<.001) and perceived relevance of telehealth after the pandemic (*P*=.008) but not in telehealth interest (*P*=.09) and perceived importance of telehealth in education (*P*=.09). Details on pairwise comparisons between the different subgroups are described in Multimedia Appendix 3. For each item, smaller values indicate better (more positive) agreement. In summary, significant pairwise differences were observed mainly for the study programs, specifically when comparing the ratings regarding telehealth knowledge (HAE<ORT, HAE<DIE, HAE<ANE, HAE<MID, HAE<NUR, SLT<MID, SLT<NUR, OT<MID, OT<NUR, PT<MID, and PT<NUR), telehealth importance (HAE<PT, HAE<MID, and SLT<MID), and the postpandemic role of telehealth (ANP<PT, ANP<MID, and ANP<ORT). For gender, the null hypotheses were rejected by the overall Kruskal Wallis tests for all 4 domains, and thus, no subsequent pairwise comparisons were conducted. For generations, the only significant pairwise comparison was for the role after the pandemic, where Generation Z had more positive ratings than Generation Y. For study progress, the only significant pairwise comparison was for the role after the pandemic, where the first 2 master’s semesters had more positive ratings than the fifth to sixth bachelor’s semesters.

**Table 3:** Results of the Kruskal Wallis H test for each subgroup test.

	Telehealth interest		Telehealth knowledge		Telehealth importance in education			Telehealth relevance after pandemic	
	Kruskal Wallis H test (*df*)	*P* value	Kruskal Wallis H test (*df*)	*P* value	Kruskal Wallis H test (*df*)	*P* value	Kruskal Wallis H test (*df*)		*P* value
Study programs	25.3 (10)	.005	70.6 (10)	<.001	33.0 (10)	<.001	25.8 (10)		.004
Genders	0.9 (2)	.63	3.3 (2)	.19	0.6 (2)	.73	1.2 (2)		.55
Age and generation	4.0 (2)	.14	3.3 (2)	.19	8.8 (2)	.01	5.5 (2)		.06
Semester	8.2 (4)	.09	43.1 (4)	<.001	8.0 (4)	.09	13.7 (4)		.008

### Telehealth Interest

**Figure 4:**
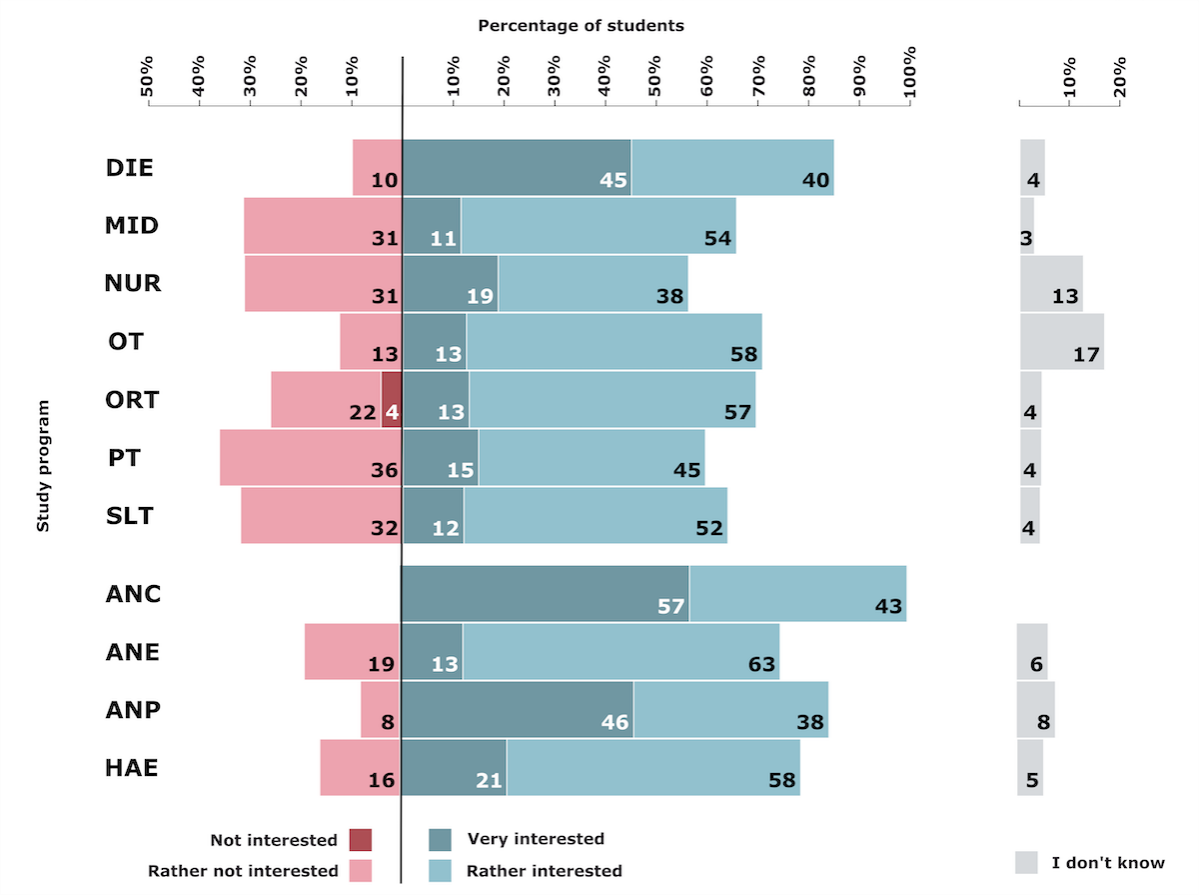
Students’ interest in telehealth based on the study program. ANC: advanced nursing counseling; ANE: advanced nursing education; ANP: advanced nursing practice; DIE: dietetics; HAE: health assisting engineering; MID: midwifery; NUR: health care and nursing; ORT: orthoptics; OT: occupational therapy; PT: physiotherapy; SLT: speech and language therapy.

Overall, 19.5% (51/261) of the students were very interested and 49.4% (129/261) of the students were rather interested in telehealth. Study programs with the highest interest ratings (very or rather interested) were ANC (7/7, 100%), DIE (17/20, 85%), and ANP (11/13, 84%). Moreover, 24.1% (63/261) of students were less interested and 0.4% (1/261) were not interested in telehealth. The study programs with the most uninterested students (rather not or not interested) were PT (17/47, 36%), SLT (8/25, 32%), MID (11/35, 31%), and NUR (10/32, 31%). The percentages by study program are presented in Figure 4.

### Telehealth Knowledge

**Figure 5:**
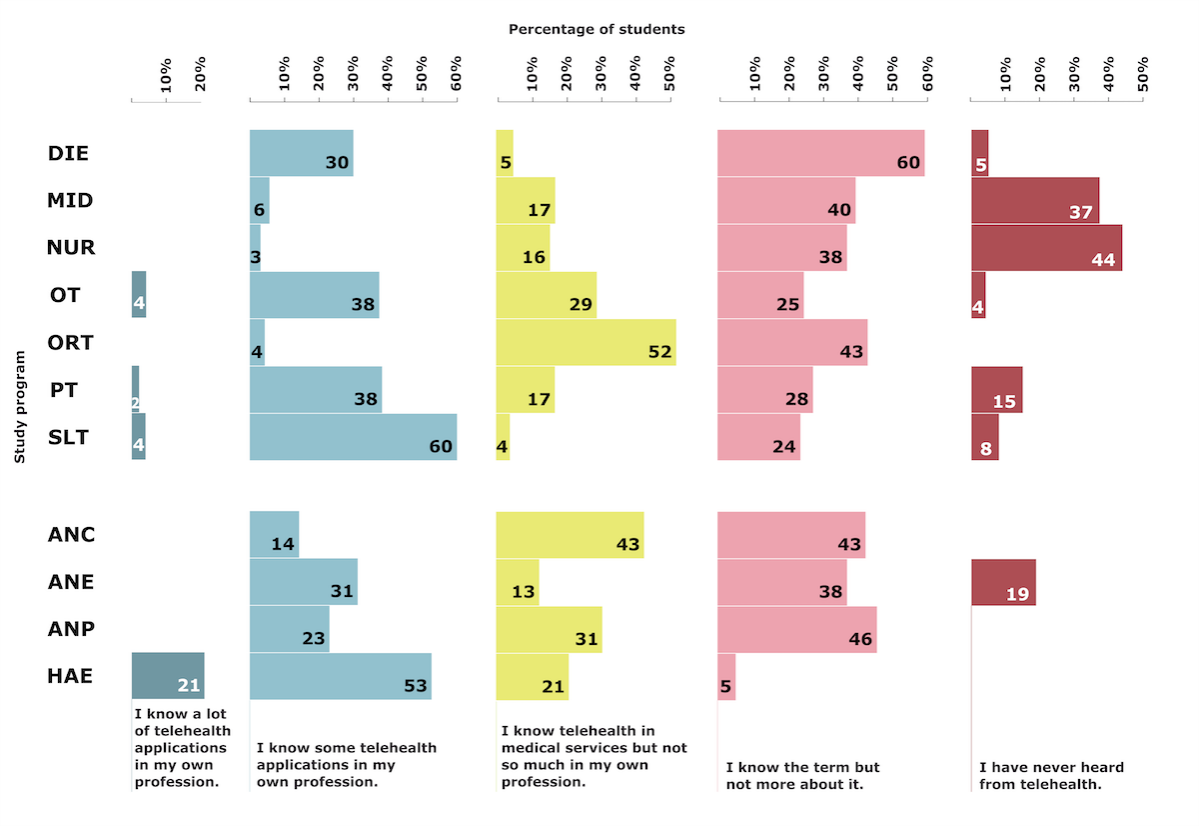
Students’ self-assessed knowledge about telehealth based on the study program. ANC: advanced nursing counseling; ANE: advanced nursing education; ANP: advanced nursing practice; DIE: dietetics; HAE: health assisting engineering; MID: midwifery; NUR: health care and nursing; ORT: orthoptics; OT: occupational therapy; PT: physiotherapy; SLT: speech and language therapy.

Only 2.7% (7/261) of the students stated that they have already dealt intensively with telehealth in their own profession and that they knew a lot of applications, 27.2% (71/261) stated that they knew some telehealth applications in their own profession, 20.3% (53/261) stated that they knew telehealth in medical services but not in their own profession, 34.1% (89/261) stated that they knew the term but nothing more about it, and 15.7% (41/261) had never heard of telehealth. The percentages by study program are presented in Figure 5.

### Telehealth Importance in Education

**Figure 6:**
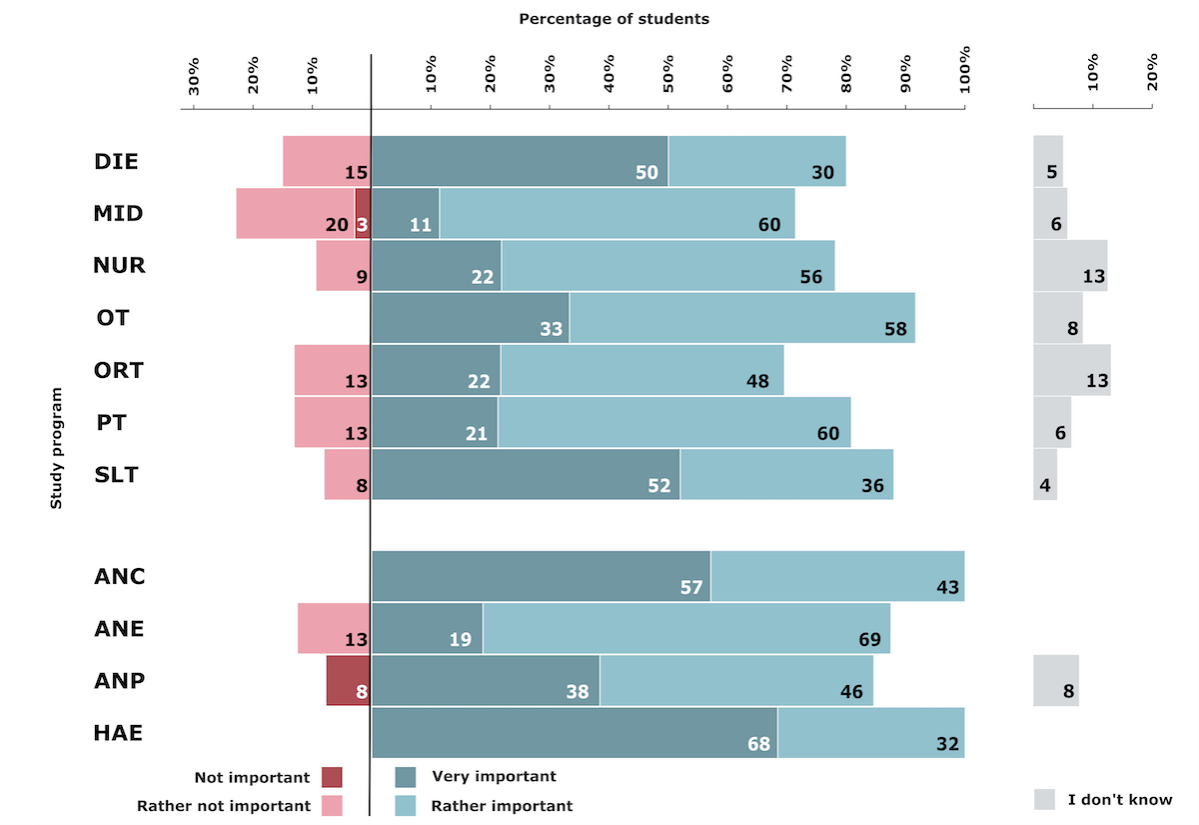
Students’ perceived importance of telehealth in education. ANC: advanced nursing counseling; ANE: advanced nursing education; ANP: advanced nursing practice; DIE: dietetics; HAE: health assisting engineering; MID: midwifery; NUR: health care and nursing; ORT: orthoptics; OT: occupational therapy; PT: physiotherapy; SLT: speech and language therapy.

Overall, 31.4% (82/261) of the students thought telehealth was very important for their education, and 50.9% (133/261) of the students rated it as rather important. The study programs with the highest importance ratings (very or rather important combined) were ANC (7/7, 100%), HAE (19/19, 100%), and OT (22/24, 91%). Moreover, 10% (26/261) of the students thought it was rather not important, and 1.1% (3/261) thought it was not important. The highest percentages of unimportance ratings (not or rather not important) were in MID (8/35, 23%), DIE (3/20, 15%), and ORT (4/23, 13%), PT (3/47, 13%), and ANE (1/13, 13%). The percentages by study program are depicted in Figure 6.

### Telehealth Relevance After the Pandemic

**Figure 7:**
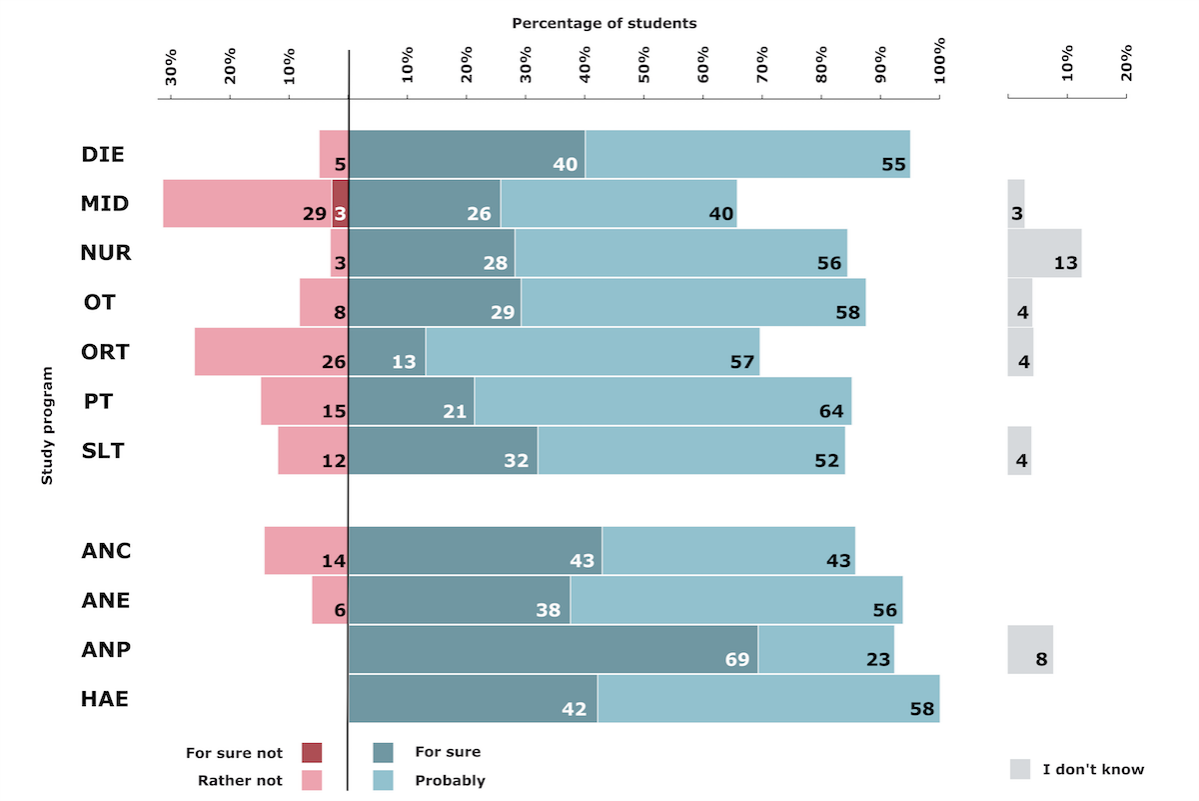
Students’ perceived relevance of telehealth after the pandemic. ANC: advanced nursing counseling; ANE: advanced nursing education; ANP: advanced nursing practice; DIE: dietetics; HAE: health assisting engineering; MID: midwifery; NUR: health care and nursing; ORT: orthoptics; OT: occupational therapy; PT: physiotherapy; SLT: speech and language therapy.

Overall, 30.7% (80/261) of the students thought that telehealth will, for sure, be relevant in their profession after the pandemic, and 53.3% (139/261) of the students thought that telehealth would probably be relevant in their profession. The study programs that rated the future relevance of telehealth as highest were HAE (19/19, 100%), DIE (19/20, 95%), and ANE (15/16, 94%). Furthermore, 12.3% (32/261) of the students stated that it will rather not be relevant and 3.4% (9/261) stated that it will for sure not be relevant. The study programs that least anticipated a future relevance of telehealth were MID (11/35, 32%), ORT (6/23, 26%), and PT (7/47, 15%). The percentages by study program are depicted in Figure 7.

### Relevance of Different Forms of Telehealth Provision

**Figure 8:**
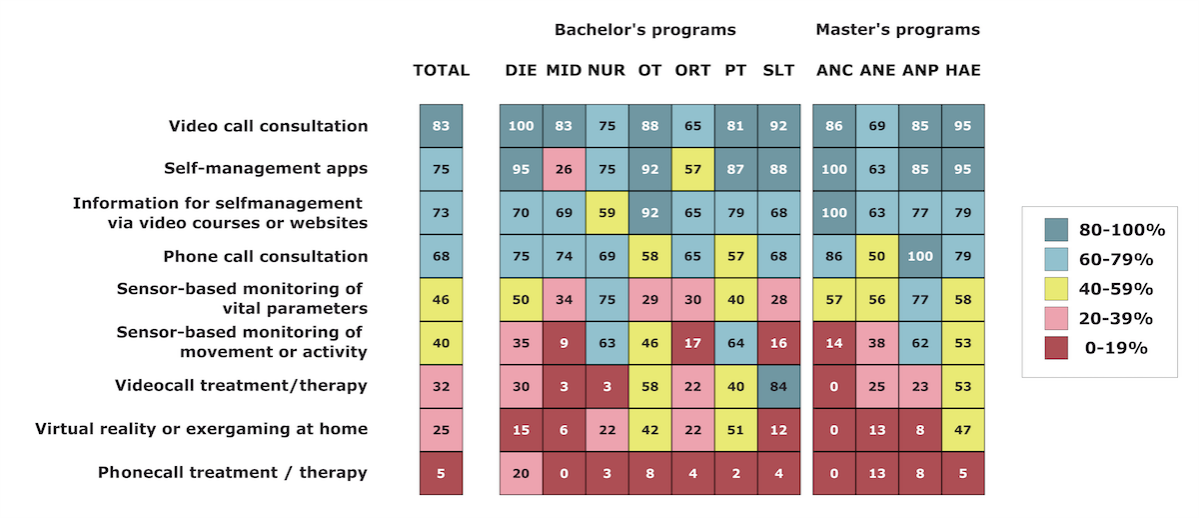
Students’ perception of the relevance of different forms of telehealth concerning their own profession (the percentage of students that believes this telehealth form is relevant in their profession). ANC: advanced nursing counseling; ANE: advanced nursing education; ANP: advanced nursing practice; DIE: dietetics; HAE: health assisting engineering; MID: midwifery; NUR: health care and nursing; ORT: orthoptics; OT: occupational therapy; PT: physiotherapy; SLT: speech and language therapy.

The given relevance of different forms of telehealth provision in their own profession was confirmed as follows: video call consultation, 82.8% (216/261); apps for self-management, 75.1% (196/261); information for self-management via video courses or websites, 72.8% (190/261); phone call consultation, 68.2% (178/261); sensor-based monitoring of vital parameters, 46% (120/261); sensor-based monitoring of movement or activity, 39.8% (104/261); video call treatment or therapy, 32.2% (84/261); virtual reality or exergaming at home, 25.3% (66/261); and phone call treatment or therapy, 5.4% (14/261). The details of the study program are shown in Figure 8.

### Telehealth Experience

**Figure 9:**
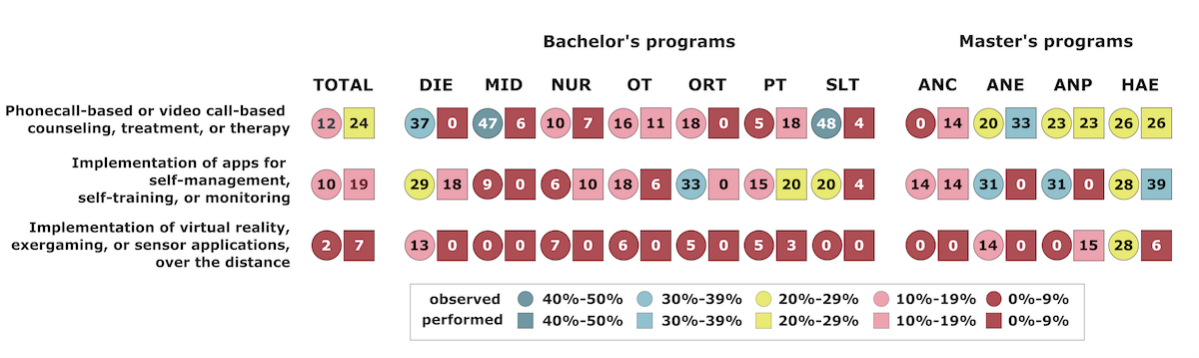
Students’ telehealth experience. The circles represent the percentage of students that have observed this form of telehealth, the squares represent the percentage of students that have performed this form of telehealth. ANC: advanced nursing counseling; ANE: advanced nursing education; ANP: advanced nursing practice; DIE: dietetics; HAE: health assisting engineering; MID: midwifery; NUR: health care and nursing; ORT: orthoptics; OT: occupational therapy; PT: physiotherapy; SLT: speech and language therapy.

Overall, 45.6% (119/261) of the students already had telehealth experience. Furthermore, 22.2% (58/261) of the students had observed and 10.7% (28/261) of the students had performed phone call–based or video call–based counseling, treatment, or therapy; 17.2% (45/261) of the students had observed and 9.2% (24/261) of the students had performed the implementation of apps for self-management and self-training of monitoring; and 5.7% (15/261) of the students had observed and 1.5% (4/261) of the students had performed the implementation of virtual reality, exergaming, or sensors over the distance. The details of the study program are shown in Figure 9.

### Telehealth Content Within the Curriculum

**Figure 10:**
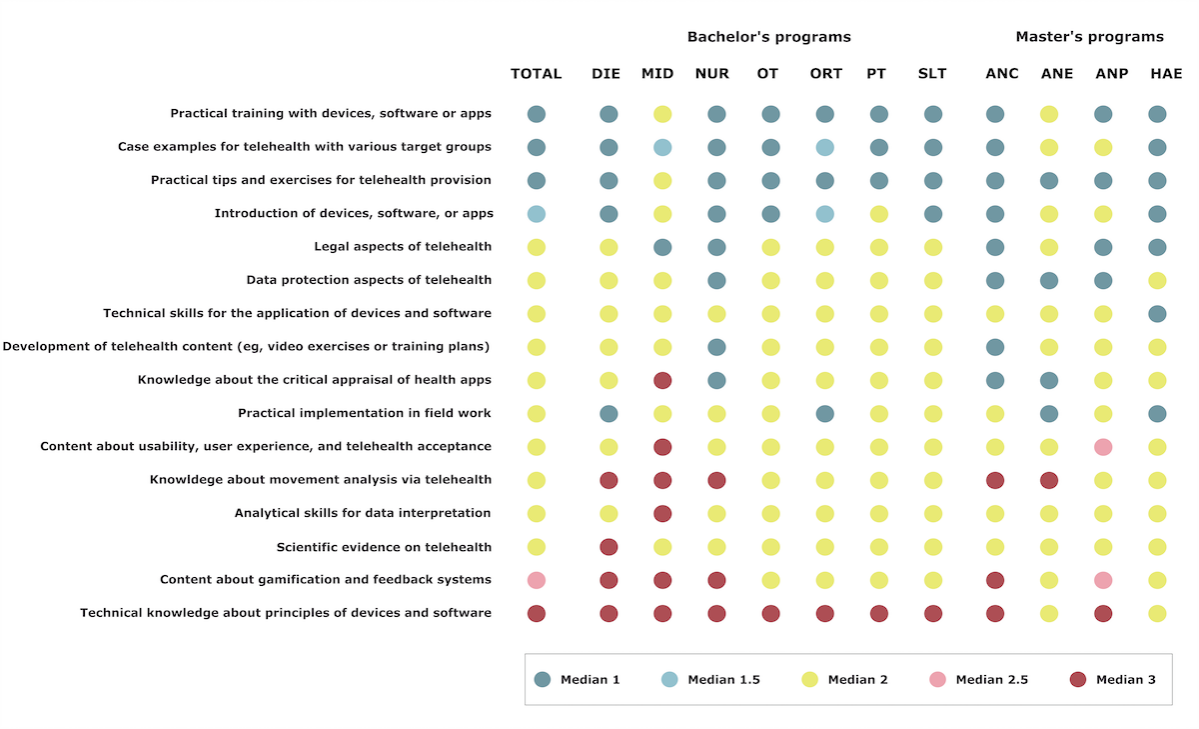
Students’ preferences for telehealth content based on the study program. The circles represent the median of all answers with 1=for sure, 2=rather yes, 3=rather not, or 4=for sure not. ANC: advanced nursing counseling; ANE: advanced nursing education; ANP: advanced nursing practice; DIE: dietetics; HAE: health assisting engineering; MID: midwifery; NUR: health care and nursing; ORT: orthoptics; OT: occupational therapy; PT: physiotherapy; SLT: speech and language therapy.

Students’ preferences for telehealth content within their curriculum from highest to lowest ranking were practical training with devices, software, or apps (median 1), case examples for telehealth with various target groups (median 1), practical tips and exercises for telehealth provision (median 1), introduction of devices, software or apps (median 1.5), legal aspects of telehealth (median 2), data protection aspects of telehealth (median 2), technical skills for the application of devices and software (median 2), development of telehealth content (eg, video exercises or training plans; median 2), knowledge about the critical appraisal of health apps (median 2), practical implementation in field work (median 2), content about usability, user experience, and telehealth acceptance (median 2), knowledge about movement analysis via telehealth (median 2), analytical skills for data interpretation (median 2), scientific evidence on telehealth (median 2), content about gamification and feedback systems (median 2.5), and technical knowledge about principles of devices and software (median 3). Details by the study program are shown in Figure 10.

Overall, 21.8% (55/252) of the students preferred to learn about telehealth with students in their study program, 10% (25/252) preferred interdisciplinary courses, 60.7% (153/252) preferred both of them, 3.2% (8/252) did not want to learn about telehealth at all, and 4.4% (11/252) did not know. Furthermore, 30.6% (77/252) of the students wanted telehealth to be taught within required subjects, 62.3% (157/252) wanted telehealth to be taught within elective subjects, 1.6% (4/252) thought it should not be incorporated into the curriculum, and 5.6% (14/252) did not know. In bachelor’s programs, 10.5% (20/190) of the students preferred the first or second semester, 60.5% (115/190) preferred the third or fourth semester, 26.8% (51/190) preferred the fifth or sixth semester, and 2.1% (4/190) of participants preferred none of them. Overall, 30% (13/44) of the master’s students thought that the first or second semester and 68% (30/44) thought that the third or fourth semester would be most appropriate, and 1 (N=1, 2%) student felt that none was appropriate.

## Discussion

### Overview

Our study provides novel insight into the telehealth knowledge, skills, attitudes, and experience of health care and nursing students and its potential integration into health care and nursing education and practice. The results suggest that there is substantial interest in telehealth among health care and nursing students but a lack of knowledge and experience with it. We discovered similarities and differences among various student groups, which will be discussed in detail and with regard to previously proposed telehealth competency frameworks for health care professionals.

### Telehealth Interest

There was a generally high level of interest in telehealth across all study programs. The study programs with the highest median interest in telehealth were 3 master’s programs (ANC, ANP, and HAE) and 1 bachelor’s program (DIE). Interest in telehealth appears to be higher among master’s students than among bachelor’s students, possibly because of their advanced level of education and experience. Students in master’s programs may have gained more professional working experience, which could have raised their awareness of the potential benefits of telehealth, such as increasing access to care [43], improving patient outcomes [44], and reducing health care costs [45]. Moreover, they might have encountered that telehealth has not yet become a ubiquitous component of the health care system. Furthermore, health care master’s programs often place greater emphasis on leadership and innovation, which could make students more interested in exploring new methods [46]. Students in master’s programs may be more focused on career advancement opportunities and recognize the potential of telehealth to create new roles or expand existing ones in the health care sector. This result also suggests that higher education may play an important role in promoting the adoption and use of telehealth in health care.

However, the students of the bachelor’s programs displayed a substantial level of interest in telehealth, which remained consistent across various semesters and generations. The slight differences in interest between the study programs could be because of differences in their professions and the extent to which telehealth is currently integrated into their practices. For example, dietitians may have a stronger focus on counseling and patient education without manual or physical approaches compared with other professions, such as MID, NUR, PT, and OT. Although these professions also have an important educational role, their physical nature may make in-person consultations more essential for their professions, whereas for DIE, telehealth consultations may be a more practical and effective option. In addition, SLT has a strong focus on communication and telehealth, especially in the form of synchronous videoconferencing, and has been successfully used for several years, for example, in rural and remote areas of countries such as Australia and Canada [47].

The high level of interest in telehealth among health care students shows a positive attitude toward the technology, indicating that many perceive it as a beneficial tool in their future professional practice. However, enthusiasm alone is not sufficient for effective telehealth practice; it must be supplemented by the right competencies as well as the ability to demonstrate successful performance in using telehealth tools and services.

### Telehealth Knowledge and Experience

In general, most students showed some level of familiarity with telehealth, with only a small number of students reporting that they had never heard of it. However, there appears to be a profound lack of specific telehealth knowledge, profession-specific applications, and telehealth experience. Moreover, there were significant differences between the professions in terms of the level of knowledge about telehealth. For example, students from the bachelor’s programs PT, OT, and SLT and the master’s program HAE reported the highest levels of knowledge about telehealth applications in their professions. We can see that in some professions most students had never heard of telehealth or only knew the term but nothing more about it (applies to DIE, MID, NUR, and ANE). While in 3 of the 4 master’s programs all students at least knew the term telehealth, this was not the case for 6 of the 7 bachelor’s programs.

A relatively low percentage of students already had experience with telehealth applications. In Austria, health care professionals have worked mostly in face-to-face settings, but the COVID-19 pandemic had a significant influence on the attitudes toward telehealth service provision and its implementation [9,48]. Nevertheless, the findings of this study suggest that especially bachelor’s students have only rarely come into touch with it. However, increased exposure to telehealth in academic settings and practical experience are important to enhance awareness and adoption of this emerging health care approach [49]. Only 35.4% (86/243) of all students reported direct or indirect experience with phone or video call–based counseling, treatment, or therapy; 29.5% (69/234) had implemented apps for self-management, self-training, or monitoring; and only 8.3% (19/229) had experience with the implementation of virtual reality, exergaming, or sensors over a distance. However, these percentages vary among different health care profession students. The highest percentage of students with experience in phone or video call–based counseling, treatment, or therapy were studying the bachelor’s programs SLT, MID, and DIE and the master’s programs ANE, ANP, and HAE. While master’s students usually already have gained professional experience and thus determine their chosen methods themselves, the experience of bachelor’s students mostly is limited to practical training within the curriculum and placements. Therefore, their experience with telehealth methods highly depends on their implementation by their teachers and supervisors. However, it remains unclear why some professions have had greater exposure to telehealth among their students than others. It is possible that the urgency of certain cases, particularly those related to acute therapy, led to greater adoption of telehealth in some professions. Another explanation could be that some professions, such as MID and DIE, might already have used telephone consultations more often before the pandemic than other professions. Students in the HAE program had the highest percentage of experience in implementing apps for self-management, self-training, or monitoring, and the implementation of virtual reality, exergaming, or sensors over a distance. This is not surprising as this program has a focus on health care technology and students might have an affinity for integrating more complex technologies into their practice.

### Telehealth Attitudes

Most students in all health care programs expected that telehealth will play an important role in their profession also after the pandemic. This aligns with current research that supports the future relevance of telehealth in health care [5,50-52]. The overall high rates of expectations among health care students regarding the integral role of telehealth in the future of health care emphasizes the need for a stronger integration of telehealth education into health care curricula. Previous surveys in other countries also found a strong belief of students that telehealth services will be strongly integrated in the future [49,52]. Approximately 30% of the participants from MID (11/35) and ORT (6/23) form an exemption and believed that telehealth will not or rather not be relevant in their profession after the pandemic. In ORT, in particular, telehealth practices may not yet be as established as in other health care professions. Similar to other professions, orthoptists heavily rely on hands-on procedures, but they may require even more specialized equipment than others for the assessment and treatment of eye disorders.

Video call consultations seem to be the most widely accepted form of telehealth provision, with a high percentage of students from all study programs agreeing that they have an important role in their profession. Students may recognize the benefits of video integration for observations, capturing interpersonal features, nonverbal cues, and eye contact that may be lost when relying solely on phone calls [51]. Moreover, in recent years, videoconferencing has seen substantial growth across various aspects of daily life, driven by technological advancements, faster internet speeds, and the transition to remote work, particularly during the COVID-19 pandemic [53]. Hence, it is conceivable that students can best envision the use of video calls within the scope of telehealth, as they are familiar with this technology from other contexts.

Self-management apps are also widely accepted, with the exception of MID, which has noticeably lower scores. This is in line with previous research that reported that 58% of the surveyed nonphysicians (including MID) categorically rejected self-monitoring apps in pregnancy [54]. Self-management apps hold potential value for a wide range of contexts and stakeholders, including patients, health care professionals, and caregivers [55] and should therefore be incorporated into education [56]. In addition, the provision of information for self-management via video courses or websites was perceived as relevant by a majority of the students. Moreover, simple phone call consultations were perceived as more relevant compared with treatments or therapy over the phone, which received more skepticism among the surveyed students. This appears to indicate students’ uncertainty regarding the feasibility or effectiveness of implementing specific interventions, techniques, or procedures through virtual means. Sensor-based monitoring of vital parameters is relatively well accepted among students in NUR and those in master’s programs. Compared with the other students examined, students in NUR likely already had the most frequent experience with monitoring vital parameters in their current roles and constitute a significant portion of patient care. Consequently, students may perceive a direct potential for alleviating their workload in their professional lives through remote monitoring technologies. Virtual reality or exergaming at home is not widely accepted among students, although it is most accepted among students in the OT, PT, and HAE programs. Exergaming interventions have demonstrated effectiveness in enhancing balance, function, physical activity levels, strength, fatigue, emotions, cognition, and pain relief [57]. Consequently, these interventions hold relevance for professionals and students in related fields.

There was a high level of agreement among students in all study programs that telehealth is important for their education. Only a minority of students of MID and ANP programs thought that this was not important. However, curricula for health care professionals have not yet widely incorporated telehealth [26] and are not consistent in their educational approaches [31]. However, health educators have started to recommend or plan to incorporate telehealth into the curriculum [58]. Furthermore, research is being conducted on which telehealth competencies should be implemented in education and with what didactic means [16,31,59-61].

Students expressed a strong desire for practical training that included hands-on experience with telehealth devices, software, and apps; case examples for telehealth with various target groups; and practical tips and exercises for telehealth provision. Previously published telehealth curricula had similarly presented a strong focus on practical experience [61]. As with any new technology or practice, students often benefit from experiential learning and simulation [62]. Therefore, it is reasonable for inexperienced health care students to prioritize practical training with devices, software, or apps; case examples for telehealth with various patient groups; and practical tips and exercises for telehealth provision. This is in line with a prior study reporting that new graduate physiotherapists perceived exposure to and practical skills training for telehealth as essential for their profession [27]. Another study with new graduate speech and language therapists concluded that they should learn to initiate telepractice service delivery through demonstration and role play to reduce initial anxieties [63].

The students’ preference for both interdisciplinary and program-specific courses might be because telehealth is a complex and multidisciplinary field that requires a broad range of knowledge and skills [5,31] but still has profession-specific requirements and applications. Previous research has shown that students benefit from an interprofessional telehealth course [60]. The main reason for preferring electives could be that students want the flexibility to choose courses that align with their specific interests and career goals. Moreover, as mentioned by some students with additional comments, the curricula and timetables are already very intense and dense. Students might fear that the introduction of new content into the curriculum would come at the expense of other relevant study content. On the other hand, the preference for compulsory subjects by 30.6% (77/252) of the students could be because students feel that telehealth is an important topic that should be incorporated into the core curriculum of their program. Most bachelor’s students had a preference for learning about telehealth in the third or fourth semester. Master’s students also showed a slight preference for telehealth content in the second part of their education. This could be because students have already acquired a foundational knowledge of health care by this time and are better equipped to understand the complex nature of telehealth. In contrast, students in their first or second semester may be overwhelmed with this topic and may not have the necessary foundational knowledge to fully comprehend the nuances of telehealth.

### Implications for Telehealth Education

Given the observed high interest and mainly positive attitude, but relatively low levels of perceived knowledge, and experience in telehealth, we conclude that it is important to enhance telehealth education for health care and nursing students. The apparent divide between perceived telehealth competence and importance of telehealth underscores the necessity that telehealth education should be integrated into the core curriculum, despite students having a preference for elective courses when directly asked. On the basis of the limited availability of publicly funded, profession-specific master’s programs in Austria [64], we believe that it is important to integrate basic telehealth education at the bachelor’s level to reach as many students as possible. However, this might not be applicable to countries with a different educational structure. Curricula should strategically incorporate the principles of Miller pyramid of clinical competence into telehealth education by emphasizing competency across the levels of knowledge, skills, performance, and action and by providing opportunities to form attitudes, as highlighted by other authors [16,60]. We suggest that there is a need for increased knowledge transfer, practical exposure, and training in the use of telehealth applications, especially in professions with lower levels of knowledge about telehealth, to increase their awareness and understanding of the potential benefits of telehealth, their specific skills, and therefore overall competency in their respective fields. It is further crucial to empower educators with the necessary competencies to effectively teach telehealth and to provide organizational framework conditions to integrate telehealth into the curricula [65].

As the students preferred to learn about case examples and hands-on experience with devices, software, and apps used in telehealth, we suggest that they early on can become more familiar and comfortable with using them. Case examples for telehealth with various target groups can help students understand the diverse needs of different patient populations and learn how to adapt their approach accordingly. Practical tips and exercises for telehealth provision can also help students to develop skills and confidence in their ability to provide telehealth services; improve their overall competency; and understand the ethical, clinical, and legal aspects that arise when using them. Furthermore, courses should expand on the essential knowledge details of legal aspects, data protection, technical skills, critical appraisal, and scientific evidence based on or in combination with practical examples. Even if these aspects did not rank highest in the needs analysis, students confirmed their relevance. Students need to build knowledge about the legal framework in which they will operate, the importance of protecting patient data and how to maintain data privacy, and the potential risks and liabilities involved. As telehealth relies heavily on technology, students need to have the technical skills to use and troubleshoot various telehealth tools and platforms [66]. Furthermore, students need to be able to apply clinical reasoning in a telehealth context and critically appraise the scientific evidence on telehealth, including its benefits and limitations, to make informed decisions about its use [67].

In terms of content, we conclude that future telehealth curricula should focus on teaching the basics and the application of practical training on consultation over the phone with or without video integration, the integration of self-management apps, and the development or integration of video courses or websites for self-management within all study programs. This focus has been previously suggested for nurse practitioner training [5]. Furthermore, specific courses for therapeutical professions (SLT, OT, and PT) could teach the possibilities of direct therapy approaches through video calls and further exergaming and virtual reality. Sensor-based monitoring of vital parameters, movement, and activity might be more appropriate for NUR, PT, and OT students, within specialization courses for students of other bachelor’s programs that are interested in this topic, and for master’s programs. Guidelines that are specific to each profession and report on implementation, financial, and technical considerations [68] should also be integrated into the development of curricula. For instance, incorporating strategies for executing telehealth practices in fields such as OT [69], musculoskeletal physiotherapy [70], SLT [71], and nursing [72] can be beneficial.

### Limitations

This study has several limitations that impact the interpretation of the results. The sample size of 261, representing a small segment of eligible health care students, and the overall low (11%) and variable response rate across programs, may affect the results’ generalizability and comparability and raises the possibility of nonresponse bias, whereby the views of those who did not participate may systematically differ from those who did. This issue poses the risk of over- or underestimation of the true distribution of perceived telehealth competencies in the target population. In addition, the cross-sectional design, capturing attitudes at a single point in time, further limits the findings. The generation distribution differed between bachelor’s and master’s programs, which may confound the perceived importance of telehealth education, knowledge levels, and postpandemic telehealth relevance across generations. Although a large portion (151/206, 73.3%) of students in bachelor’s programs belonged to Generation Z, master’s programs had a higher representation of Generation Y, Generation X, and baby boomers (49/55, 89%). Therefore, the statistical differences in the perceived importance of telehealth education between generations and differences in telehealth knowledge and perceived relevance of telehealth after the pandemic must be interpreted with caution. It should also be noted that health professionals pursuing a master’s degree later in their careers might show more interest in innovation, making these results less generalizable to other health professionals of the same generations. Regarding the statistical analysis, it should be mentioned that multiple comparisons increase the risk of type I errors. Even with the Bonferroni adjustment, which is conservative, there is a tradeoff with statistical power, potentially leading to type II errors [73]. In addition, self-reported measures of telehealth interest and knowledge may be influenced by social desirability bias or inaccurate self-assessment. Furthermore, it was not possible to directly assess student’s telehealth skills and actual performance using a web-based survey. In addition, the study’s context, focused on students from specific health care programs in 1 Austrian university, restricts the applicability of the findings to other institutions or countries. Finally, a limitation of this study is the potential impact of the COVID-19 pandemic on the participants’ attitudes, experiences, and perspectives toward telehealth. The students who participated in this study in May 2022 were probably affected by the pandemic in various ways, including disruptions in their placements and the rapid adoption of telehealth services in health care settings. As a result, their views on telehealth might be influenced by the unique circumstances of the pandemic, which could limit the generalizability of the findings to other periods.

### Recommendations for Further Research

Future research should consider several steps to build on this study. First, expanding the study to include a larger, more diverse sample of health care students from different institutions and countries will allow for examining potential variations in knowledge, skills, performance, action, and attitudes in telehealth. In addition, exploring factors that may act as barriers or facilitators to the adoption of telehealth within health care education, such as the interest, skills, and knowledge of educators, technological infrastructure, legal and ethical considerations, or institutional barriers, is crucial. Second, conducting more intervention-based studies that aim at improving telehealth knowledge, competence, and interest among health care students [74,75] will be valuable for investigating the effectiveness of different teaching methods and content that can help identify the most effective strategies for telehealth education. Moreover, conducting longitudinal research would enable tracking changes in students’ attitudes, knowledge, and interest in telehealth over time as they progress through their education, providing a comprehensive understanding of the development and potential factors influencing these perspectives, especially in the time after the COVID-19 pandemic. Assessing the impact of telehealth training on clinical practice is important. Investigating the relationship between telehealth training during health care education and its application in clinical practice, as well as evaluating the impact of telehealth knowledge and competence on patient outcomes and health care delivery, can provide valuable insights. Finally, examining the role of interprofessional collaboration in telehealth education and practice and its impact on students’ attitudes and knowledge regarding telehealth is essential [76]. Evaluating the effectiveness of interdisciplinary courses in fostering collaboration and improving telehealth competence among health care students can contribute to the development of more efficient telehealth education strategies.

### Conclusions

Our study findings underscore the need for structured telehealth education within health care curricula to equip students with the necessary competencies for future practice. Students recognize the importance of telehealth in their future profession and feel that they need to be adequately prepared. However, the study also revealed that the level of telehealth experience and knowledge among participating health care students is currently low. Therefore, there is an urgent need to provide comprehensive telehealth education and training to health care students to prepare them for the future demands in their profession. By incorporating telehealth education into health care curricula, institutions can better prepare students for the evolving landscape of health care and promote the successful integration of telehealth into future practice.

## Appendix

Multimedia Appendix 1 Original questionnaire (German version).

Multimedia Appendix 2 Translated questionnaire (English version).

Multimedia Appendix 3 Pairwise comparisons.

## Abbreviations

ANC: advanced nursing counseling
ANE: advanced nursing education
ANP: advanced nursing practice
CROSS: Consensus-Based Checklist for Reporting of Survey Studies
DIE: dietetics
HAE: health assisting engineering
MID: midwifery
NUR: health care and nursing
ORT: orthoptics
OT: occupational therapy
PT: physiotherapy
SLT: speech and language therapy

## Data Availability

The data sets generated during or analyzed during this study are available from the corresponding author upon reasonable request.
