# Correction: Telehealth Education in Allied Health Care and Nursing: Web-Based Cross-Sectional Survey of Students’ Perceived Knowledge, Skills, Attitudes, and Experience

**DOI:** 10.2196/59919

**Published:** 2024-04-26

**Authors:** Lena Rettinger, Peter Putz, Lea Aichinger, Susanne Maria Javorszky, Klaus Widhalm, Veronika Ertelt-Bach, Andreas Huber, Sevan Sargis, Lukas Maul, Oliver Radinger, Franz Werner, Sebastian Kuhn

**Affiliations:** 1 Health Assisting Engineering FH Campus Wien University of Applied Sciences Vienna Austria; 2 Institute of Digital Medicine Philipps-University & University Hospital of Giessen and Marburg Marburg Germany; 3 Competence Center INDICATION FH Campus Wien University of Applied Sciences Vienna Austria; 4 Logopedics – Phoniatrics - Audiology FH Campus Wien University of Applied Sciences Vienna Austria; 5 Physiotherapy FH Campus Wien University of Applied Sciences Vienna Austria; 6 Occupational Therapy FH Campus Wien University of Applied Sciences Vienna Austria; 7 Orthoptics FH Campus Wien University of Applied Sciences Vienna Austria; 8 Midwifery FH Campus Wien University of Applied Sciences Vienna Austria; 9 Competence Center Nursing Sciences FH Campus Wien University of Applied Sciences Vienna Austria

In “Telehealth Education in Allied Health Care and Nursing: Web-Based Cross-Sectional Survey of Students’ Perceived Knowledge, Skills, Attitudes, and Experience” (JMIR Med Educ 2024;10:e51112) an error was noted.

In the title, the word “student’s” has been revised to “students’”.

Therefore, the original title:

Telehealth Education in Allied Health Care and Nursing: Web-Based Cross-Sectional Survey of Student’s Perceived Knowledge, Skills, Attitudes, and Experience

Has been revised to:

Telehealth Education in Allied Health Care and Nursing: Web-Based Cross-Sectional Survey of Students’ Perceived Knowledge, Skills, Attitudes, and Experience

The correction will appear in the online version of the paper on the JMIR Publications website on April 26, 2024 together with the publication of this correction notice. Because this was made after submission to PubMed, PubMed Central, and other full-text repositories, the corrected article has also been resubmitted to those repositories.

