# Supplementary material for: Telehealth Education in Allied Health Care and Nursing: Web-Based Cross-Sectional Survey of Students’ Perceived Knowledge, Skills, Attitudes, and Experience
Source: JMIR Med Educ. 2024 Mar 21;10:e51112. doi: 10.2196/51112 (PMC10995793; doi:10.2196/51112)
Supplement: Multimedia Appendix 1 [file mededu_v10i1e51112_app1.pdf]

Telehealth in der Lehre

Herzlich Willkommen zur Umfrage "Telehealth in der Lehre an der FH Campus Wien"!

Diese Umfrage findet im Rahmen des von der MA23 Stadt Wien geförderten Forschungsprojektes "Telehealth Blocks" statt, in welchem Faktoren für die erfolgreiche Implementierung von Telehealth in die Praxis und Lehre erforscht werden.

Ziel dieser Umfrage ist den Bedarf und das Interesse von Studierenden ausgewählter Gesundheitsberufe an dem Thema Telehealth zu erheben und in Folge ein maßgeschneidertes Lehrangebot zu entwickeln.

Das Ausfüllen des Fragebogens nimmt in etwa 5-10 Minuten in Anspruch.

Die Teilnahme an dieser Erhebung ist freiwillig und kann während der Beantwortung jederzeit abgebrochen werden. Die rechtliche Grundlage für die Verarbeitung bildet Ihre ausdrückliche Einwilligung.

Im Rahmen Ihrer Teilnahme werden keinerlei direkt personenbezogene Daten gespeichert. Sämtliche Auswertungen und Analysen auf Subgruppenebene, sowie deren Publikation, werden so durchgeführt, dass etwaige Rückschlüsse auf Einzelpersonen durch „Merkmalskombinationen“ ausgeschlossen sind.

Nach Beendigung und Absenden des Fragebogens kann Ihnen kein Datensatz eindeutig zugeordnet werden. Daher können in diesem Fall die Rechte betreffend Auskunft, Berichtigung, Löschung oder Einschränkung der Verarbeitung Ihrer Angaben nicht erfüllt werden.
Die im Rahmen des Projektes erhobenen Daten werden entsprechend der Förderrichtlinien der MA23 zehn Jahre nach Abschluss des Projektes gespeichert und anschließend gelöscht.

Bei Fragen zur Umfrage oder der Verarbeitung und Speicherung Ihrer Daten, wenden Sie sich bitte an die Projektleiterin Lena Rettinger unter lena.rettinger@fh-campuswien.ac.at.

Telehealth ist in der folgenden Umfrage definiert als „Erbringung von Gesundheitsdienstleistungen über die Ferne mit Hilfe von Informations- und Kommunikationstechnologien“

In dieser Umfrage sind 20 Fragen enthalten.

Studiengang

1 Studieren Sie an der FH Campus Wien? *

❗ Bitte wählen Sie eine der folgenden Antworten:
Bitte wählen Sie nur eine der folgenden Antworten aus:

- ○ Ja.
- ○ Nein.

Ein aufrechtes Studium an der FH Campus Wien ist Voraussetzung für die Umfrage-Teilnahme.

2 Welchen Studiengang besuchen Sie aktuell? *

❗ Bitte wählen Sie eine der folgenden Antworten:
Bitte wählen Sie nur eine der folgenden Antworten aus:

- ○ Advanced Nursing Counseling
- ○ Advanced Nursing Education
- ○ Advanced Nursing Practice
- ○ Diätologie
- ○ Ergotherapie
- ○ Gesundheits- und Krankenpflege
- ○ Health Assisting Engineering
- ○ Hebammen
- ○ Logopädie
- ○ Orthoptik
- ○ Physiotherapie
- ○ Ich besuche einen anderen Studiengang.

Der Besuch eines explizit angeführten Studiengangs ist Voraussetzung für die Umfrage-Teilnahme.

3 Bitte geben Sie den Bachelor-Studiengang, den Sie vor Ihrem Masterstudium besucht haben an. *

❗ Bitte wählen Sie eine der folgenden Antworten:
Bitte wählen Sie nur eine der folgenden Antworten aus:

- ○ Ergotherapie
- ○ Physiotherapie
- ○ Gesundheits- und Krankenpflege
- ○ Einen technischen Bachelorstudiengang
- ○ Sonstiges ____________

Demographische Daten

4 Geben Sie bitte Ihre Altersgruppe an. *

Bitte wählen Sie nur eine der folgenden Antworten aus:

- ○ unter 20 Jahre
- ○ 21-25 Jahre
- ○ 26-30 Jahre
- ○ 31-35 Jahre
- ○ 36-40 Jahre
- ○ 41-45 Jahre
- ○ 46-50 Jahre
- ○ über 50 Jahre

5 Geben Sie bitte Ihr Geschlecht an. *

Bitte wählen Sie nur eine der folgenden Antworten aus:

- ○ Weiblich
- ○ Männlich
- ○ Divers

6 In welchem Semester befinden Sie sich aktuell? *

Bitte wählen Sie nur eine der folgenden Antworten aus:

- ○ 1. Semester
- ○ 2. Semester
- ○ 3. Semester
- ○ 4. Semester
- ○ 5. Semester
- ○ 6. Semester

7
Bitte schätzen Sie Ihre eigene Kompetenz im Umgang mit Informations- und Kommunikationstechnologien (Computer, Smartphone, Tablet,…) auf einer Skala nach dem Schulnoten-System ein.
*

Bitte wählen Sie nur eine der folgenden Antworten aus:

- ○ 1 - sehr gut
- ○ 2 - gut
- ○ 3 - befriedigend
- ○ 4 - genügend
- ○ 5 - nicht genügend

Telehealth Fragen

8
Wie würden Sie Ihr persönliches Interesse am Thema Telehealth, in Bezug auf Ihre eigene (zukünftige) Berufsgruppe einschätzen?
*

Bitte wählen Sie nur eine der folgenden Antworten aus:

- ○ Kein Interesse.
- ○ Wenig Interesse.
- ○ Relativ großes Interesse.
- ○ Sehr großes Interesse.
- ○ Ich weiß nicht / Keine Angabe.

Telehealth ist in dieser Umfrage definiert als „Erbringung von Gesundheitsdienstleistungen über die Ferne mit Hilfe von Information- und Kommunikationstechnologien“

9
Wie würden Sie Ihren Wissensstand zum Thema Telehealth einschätzen? Bitte kreuzen Sie an, was am Besten auf Sie zutrifft.
*

Bitte wählen Sie nur eine der folgenden Antworten aus:

- ○ Ich habe noch nie von Telehealth gehört.
- ○ Ich kenne den Begriff, weiß aber sonst kaum etwas darüber.
- ○ Ich kenne Telehealth vor allem im Zusammenhang mit ärztlichen Dienstleistungen, aber wenig in Bezug auf meine eigene Berufsgruppe.
- ○ Ich kenne einige Telehealth-Anwendungen oder Anwendungsgebiete in meiner eigenen Berufsgruppe.
- ○ Ich habe mich bereits intensiv mit dem Thema Telehealth in Bezug auf meine eigene Berufsgruppe auseinandergesetzt und kenne viele unterschiedliche Anwendungen oder Anwendungsgebiete.

10
Denken Sie, dass es für Sie wichtig ist, sich in Ihrem Studium mit dem Thema Telehealth auseinanderzusetzen?
*

Bitte wählen Sie nur eine der folgenden Antworten aus:

- ○ Ja, sehr wichtig.
- ○ Ja, ein wenig wichtig.
- ○ Nein, eher nicht wichtig.
- ○ Nein, gar nicht wichtig.
- ○ Ich weiß nicht / Keine Angabe.

11
Denken Sie, dass das Thema Telehealth über die Pandemie hinaus eine wichtige Rolle für Ihre Berufsgruppe spielen wird?
*

Bitte wählen Sie nur eine der folgenden Antworten aus:

- ○ Ja, ganz sicher.
- ○ Ja, wahrscheinlich.
- ○ Nein, eher nicht.
- ○ Nein, sicher nicht.
- ○ Ich weiß nicht / Keine Angabe.

12
Welche der folgenden Anwendungen von Telehealth, spielen in Ihrer Berufsgruppe aus Ihrer Sicht eine wichtige Rolle oder werden in Zukunft eine wichtige Rolle spielen? (Mehrfachnennungen möglich)
*

Bitte wählen Sie alle zutreffenden Antworten aus:

- ☐ Beratungen über das Telefon
- ☐ Beratungen über Videotelefonie
- ☐ Behandlungen über das Telefon
- ☐ Behandlungen über Videotelefonie
- ☐ Einsatz von Apps zur Verwendung von Patient*innen außerhalb der Praxis/Gesundheitseinrichtung zum Selbstmanagement
- ☐ Einsatz von Sensoren zum Monitoring von Vitalparametern
- ☐ Einsatz von Sensoren zum Monitoring von Bewegungsparametern od. Aktivität
- ☐ Einsatz von Virtual Reality-Anwendungen oder Exergames/Serious Games außerhalb der Praxis/Gesundheitseinrichtungen
- ☐ Einsatz von Videokursen bzw. Übungsmaterial zum Selbstmanagement auf Websites
- ☐ Ich weiß nicht / Keine Angabe.
- ☐ Sonstiges: ____________

13 Haben Sie Telehealth bereits in der eigenen beruflichen Praxis oder im Berufspraktikum angewendet oder beobachtet?

Bitte wählen Sie die zutreffende Antwort für jeden Punkt aus:

	Selber durchgeführt	Beobachtet	Nicht durchgeführt oder beobachtet
**Beratung oder Therapie über Telefonie oder Videotelefonie**	○	○	○
**Einsatz von Apps zum Selbstmanagement, Eigentraining oder Monitoring über die Ferne**	○	○	○
**Einsatz von Sensorunterstützung, Exergaming oder Virtual Reality über die Ferne**	○	○	○
**Sonstige Erfahrungen im Bereich Telehealth**	○	○	○

14 Welche sonstigen praktischen Erfahrungen oder Beobachtungen konnten Sie im Bereich Telehealth bereits machen?

Bitte geben Sie Ihre Antwort hier ein:

Telehealth in der Lehre

15
Was würden Sie in Ihrem Studium gerne zum Thema Telehealth lernen?
Beurteilen Sie bitte ob Sie die einzelnen Themen sicher nicht, eher nicht, eher schon oder unbedingt erlernen bzw. üben möchten.

Bitte wählen Sie die zutreffende Antwort für jeden Punkt aus:

	Sicher nicht	Eher nicht	Eher schon	Unbedingt
**Technisches Hintergrundwissen zur Funktionsweise von Geräten oder Software**	○	○	○	○
**Technische Skills für die Anwendung von Geräten oder Software**	○	○	○	○
**Analytische Skills zur Interpretation von Daten, welche über die Ferne übertragen werden**	○	○	○	○
**Wissenschaftliche Evidenzen zu Telehealth**	○	○	○	○
**Anwendungsbeispiele bei unterschiedlichen Zielgruppen**	○	○	○	○
**Kennenlernen von verschiedenen Geräten, Software oder Apps**	○	○	○	○
**Praktisches Üben mit verschiedenen Geräten, Software oder Apps**	○	○	○	○
**Rechtliche Aspekte im Zusammenhang mit Telehealth**	○	○	○	○
**Wissen zu Datenschutz im Zusammenhang mit Telehealth**	○	○	○	○
**Praktische Tipps und Übungen zur Durchführung von Telehealth**	○	○	○	○
**Entwicklung von Telehealth-Content (z.B. Erstellen von Übungsvideos oder Plänen)**	○	○	○	○
**Analyse von Bewegungen in einer Videoübertragung**	○	○	○	○
**Inhalte zu Gamification und Feedbacksystemen**	○	○	○	○
**Inhalte zu Benutzungsfreundlichkeit, User Experience und Akzeptanz von Telehealth-Technologien**	○	○	○	○
**Inhalte zur Beurteilung der Tauglichkeit von Health-Apps**	○	○	○	○
**Praktischer Einsatz im Berufspraktikum**	○	○	○	○

16
Gibt es sonst noch etwas, das Sie gerne in Bezug auf Telehealth erlernen oder üben würden, das in der Liste nicht erwähnt wurde? Wenn ja, geben Sie hier bitte Inhalte an, die Ihnen in der Auflistung gefehlt haben.

Bitte geben Sie Ihre Antwort hier ein:

Setting

17 In welchem Setting würden Sie gerne über Telehealth lernen? *

Bitte wählen Sie nur eine der folgenden Antworten aus:

- ○ Mit Studierenden meines Studiengangs.
- ○ Interdisziplinär gemeinsam mit Studierenden anderer Studiengänge.
- ○ Sowohl im eigenen Studiengang, als auch interdisziplinär mit Studierenden anderer Studiengänge.
- ○ Gar nicht.
- ○ Ich weiß nicht / keine Angabe.

18
Wie sollte Telehealth im Curriculum verankert sein?
*

Bitte wählen Sie nur eine der folgenden Antworten aus:

- ○ Als Pflichtfach bzw. im Rahmen von anderen Pflichtfächern.
- ○ Als Wahl(pflicht)fach bzw. im Rahmen von anderen Wahl(pflicht)fächern.
- ○ Gar nicht.
- ○ Ich weiß nicht / keine Angabe.

19
Welchen Zeitpunkt im Studium halten Sie für das Thema für geeignet?
*

Bitte wählen Sie nur eine der folgenden Antworten aus:

- ○ Im oder ab dem 1. oder 2. Semester
- ○ Im oder ab dem 3. oder 4. Semester
- ○ Im oder ab dem 5. oder 6. Semester (nur bei Bachelorstudiengängen wählbar!)
- ○ Keinen
- ○ Ich weiß nicht / keine Angabe.

20
Möchten Sie noch etwas hinzufügen, das uns weiterhilft Telehealth in Zukunft bestmöglich in das Curriculum zu integrieren?

Bitte geben Sie Ihre Antwort hier ein:

Herzlichen Dank für Ihre Teilnahme und den wertvollen Beitrag zur maßgeschneiderten Erweiterung des Lehrangebots im Bereich Telehealth!

Ihre Eingaben wurden gespeichert. Sie können dieses Fenster nun schließen.

01.06.2022 – 06:00

Übermittlung Ihres ausgefüllten Fragebogens:
Vielen Dank für die Beantwortung des Fragebogens.
