# Supplementary material for: Telehealth Education in Allied Health Care and Nursing: Web-Based Cross-Sectional Survey of Students’ Perceived Knowledge, Skills, Attitudes, and Experience
Source: JMIR Med Educ. 2024 Mar 21;10:e51112. doi: 10.2196/51112 (PMC10995793; doi:10.2196/51112)
Supplement: Multimedia Appendix 2 [file mededu_v10i1e51112_app2.pdf]

TRANSLATED QUESTIONNAIRE (ORIGINAL LANGUAGE: GERMAN)

Telehealth in Education

Welcome to the survey "Telehealth in Education at the FH Campus Wien"!

This survey is conducted as part of the MA23-funded research project "Telehealth Blocks", which investigates factors for the successful implementation of telehealth in practice and education.

The aim of this survey is to determine the needs and interests of students from selected health professions on the topic of telehealth and subsequently develop a tailored curriculum.

Completing the questionnaire takes about 5-10 minutes.

Participation in this survey is voluntary and can be terminated at any time during the response process. In this case, your data will not be saved. Your explicit consent forms the legal basis for processing.

No direct personal data will be stored during your participation. All evaluations and analyses at the subgroup level, as well as their publication, are conducted in a way that individual conclusions are excluded.

After completing and submitting the questionnaire, no dataset can be clearly assigned to you. Therefore, in this case, you have your rights regarding access, correction, deletion, or restriction of processing your data cannot be fulfilled.

The data collected as part of the research project will be stored in accordance with the MA23 funding guidelines for ten years after the project's completion and then deleted.

If you have questions about the survey or the processing and storage of your data, please contact the project manager Lena Rettinger at lena.rettinger@fh-campuswien.ac.at.

In this survey, telehealth is defined as "the provision of health services from a distance using information and communication technologies."

This survey contains 20 questions.

STUDY PROGRAM

1. **Do you study at the FH Campus Wien?**

   Please select one of the following answers:
   - Yes.
   - No.

   Attending a study program at the FH Campus Vienna is a prerequisite for participating in the survey.

2. **Which study program are you currently attending?**

   Please select one of the following answers:
   - Advanced Nursing Counseling
   - Advanced Nursing Education
   - Advanced Nursing Practice
   - Dietetics
   - Occupational Therapy
   - Nursing
   - Health Assisting Engineering
   - Midwifery
   - Speech & Language Therapy
   - Orthoptics
   - Physical therapy
   - I am attending another course of study.

   Attending one of the specifically mentioned courses of study is a prerequisite for participating in the survey.

3. **Please specify the Bachelor's study pgrogram that you attended before your Master's studies.**
   - Please answer this question only if the following conditions are met: Answer to "Health Assisting Engineering" in question 2 (Which course of study are you currently attending?)
     - Please select one of the following answers:
       - Occupational Therapy
       - Physical Therapy
       - Nursing
       - A technical Bachelor's program
       - Other

DEMOGRAPHICS

4. **Please specify your age group.**

   Please select only one of the following answers:
   - under 20 years
   - 21-25 years
   - 26-30 years
   - 31-35 years

- 36-40 years
- 41-45 years
- 46-50 years
- over 50 years

5. **Please specify your gender.**

   Please select only one of the following answers:
   - Female
   - Male
   - Diverse

6. **In which semester are you currently?**

   Please select only one of the following answers:
   - 1st semester
   - 2nd semester
   - 3rd semester
   - 4th semester
   - 5th semester
   - 6th semester

7. **Please assess your own competence in handling information and communication technologies (Computer, Smartphone, Tablet,...) on a scale according to the school grade system.**

   Please select only one of the following answers:
   - 1 - very good
   - 2 - good
   - 3 - satisfactory
   - 4 - sufficient
   - 5 – insufficient

TELEHEALTH QUESTIONS

8. **How would you rate your personal interest in the topic of telehealth, in relation to your own (future) profession?**

   Please select only one of the following answers:
   - Not interested.
   - Less interested.
   - Rather interested.
   - Very interested.
   - Don't know.

   Telehealth in this survey is defined as: "Provision of healthcare services over distance with the help of information and communication technologies."

9. **How would you assess your knowledge about the topic of telehealth? Please check what best applies to you.**

Please select only one of the following answers:

- I have never heard of telehealth.
- I know the term but not more about it.
- I know telehealth in medical services but not so much about it in my own profession
- I know some telehealth applications in my own profession
- I know a lot of telehealth applications in my own profession

10. **Do you think it is important for you to engage with the topic of telehealth during your studies?**

Please select only one of the following answers:

- Yes, very important.
- Yes, rather important.
- No, rather not important.
- No, not important.
- Don't know.

11. **Do you believe that the topic of telehealth will play a significant role for your professional group beyond the pandemic?**

Please select only one of the following answers:

- For sure.
- Probably.
- Rather not.
- For sure not.
- Don't know.

12. **Which of the following applications of telehealth do you believe will play a significant role in your professional group in the future? (Multiple selections possible)**

Please select all applicable answers:

- Phonecall consultations
- Videocall consultations
- Phonecall treatment / therapy
- Videocall treatment / therapy
- Selfmanagement apps outside of clinics/healthcare facilities
- Sensor-based monitoring of vital parameters
- Sensor-based monitoring of movement or activity
- Virtual Reality or exergaming at home
- Information for selfmanagement via video courses or websites
- Don't know.
- Other: ___

13. **Have you already used or observed telehealth in your professional practice or during an internship?**

Please select the appropriate answer for each point:

	Carried out by yourself	Observed	Not carried out or observed
Consultation or therapy via phone or video call	◯	◯	◯
Use of apps for self-management, self-training, or remote monitoring	◯	◯	◯
Use of sensor support, exergaming, or virtual reality from a distance	◯	◯	◯
Other experiences in the field of telehealth	◯	◯	◯

14. **What other practical experiences or observations have you made in the field of telehealth?**

    Answer this question only if the following conditions are met:
    - You answered 'Carried out by yourself' or 'Observed' for question 13 (Experience: Have you already used telehealth in your professional practice or during an internship?)

    Please enter your answer here: ___

TELEHEALTH IN EDUCATION

15. **What would you like to learn in your studies about Telehealth? Please assess whether you want to learn about the individual topics.**

    Please select the applicable answer for each:

	For sure <u>not</u>	Rather not	Rather yes	For sure
Technical knowledge about principles of devices and software				
Technical skills for the application of devices and software				
Analytical skills for data interpretation				
Scientific evidence on telehealth				
Case examples for telehealth with various target groups				
Introduction of devices, software, or apps				

	For sure <u>not</u>	Rather not	Rather yes	For sure
Practical training with devices, software, or apps				
Legal aspects of telehealth				
Data protection aspects of telehealth				
Practical tips and exercises for telehealth provision				
Development of Telehealth content (e.g., video exercises or training plans)				
Knowledge about movement analysis via telehealth				
Content on gamification and feedback systems				
Content about usability, user experience, and telehealth acceptance				
Knowledge about the critical appraisal of health apps				
Practical implementation in field work				

16. Is there anything else you would like to learn or practice regarding telehealth that was not mentioned in the list?

If yes, please provide the content that you think was missing.
Please enter your answer here: ___

SETTING

17. In which setting would you like to learn about telehealth? Please select only one of the following answers:

- With students from my course of study.
- Interdisciplinary, together with students from other courses of study.
- Both in my own course of study, and interdisciplinary with students from other courses.
- Not at all.
- Don't know.

18. How should telehealth be anchored in the curriculum? Please select only one of the following answers:

- As a mandatory subject or within the framework of other mandatory subjects.
- As an elective subject within the framework of other elective subjects.

- Not at all.
- Don’t know.

19. At which point in your studies do you consider the topic to be appropriate?

Please select only one of the following answers:

- In or from the 1st or 2nd semester
- In or from the 3rd or 4th semester
- In or from the 5th or 6th semester (only selectable for Bachelor's programs!)
- None
- Don’t know.

20. Would you like to add something that will help us to best integrate Telehealth into the curriculum in the future?

Please enter your answer here: ___

Thank you very much for your participation and your valuable contribution!

Your input has been saved. You can now close this window.
