# Supplementary material for: Telehealth Education in Allied Health Care and Nursing: Web-Based Cross-Sectional Survey of Students’ Perceived Knowledge, Skills, Attitudes, and Experience
Source: JMIR Med Educ. 2024 Mar 21;10:e51112. doi: 10.2196/51112 (PMC10995793; doi:10.2196/51112)
Supplement: Multimedia Appendix 3 [file mededu_v10i1e51112_app3.pdf]

Telehealth Education of Healthcare Profession Students

Online Supplementary Material

Figure S1: Pairwise comparisons of telehealth interest by study program p2

Table S1: Pairwise comparisons of telehealth interest by study program p3

Figure S2: Pairwise comparisons of telehealth knowledge by study program p5

Table S2: Pairwise comparisons of telehealth knowledge by study program p6

Figure S3: Pairwise comparisons of importance of telehealth education by study program p8

Table S3: Pairwise comparisons of importance of telehealth education by study program p9

Figure S4: Pairwise comparisons of role after the pandemic by study program p11

Table S4: Pairwise comparisons of role after the pandemic by study program p12

Figure S5: Pairwise comparisons of role after the pandemic by generation p15

Table S5: Pairwise comparisons of role after the pandemic by generation p16

Figure S6: Pairwise comparisons of role after pandemic by semester p17

Table S6: Pairwise comparisons of role after pandemic by semester p18

Hypothesis Test Summary by Study Program

	Null Hypothesis	Test	Sig.[a,b]	Decision
1	The distribution of **telehealth interest** is the same across categories of **study program**.	Independent-Samples Kruskal-Wallis Test	0.05	Reject the null hypothesis.
2	The distribution of telehealth knowledge is the same across categories of study program.	Independent-Samples Kruskal-Wallis Test	<0.001	Reject the null hypothesis.
3	The distribution of importance of telehealth education is the same across categories of study program.	Independent-Samples Kruskal-Wallis Test	<0.001	Reject the null hypothesis.
4	The distribution of role after pandemic is the same across categories of study program.	Independent-Samples Kruskal-Wallis Test	0.004	Reject the null hypothesis.

a. The significance level is 0.05.

b. Asymptotic significance is displayed.

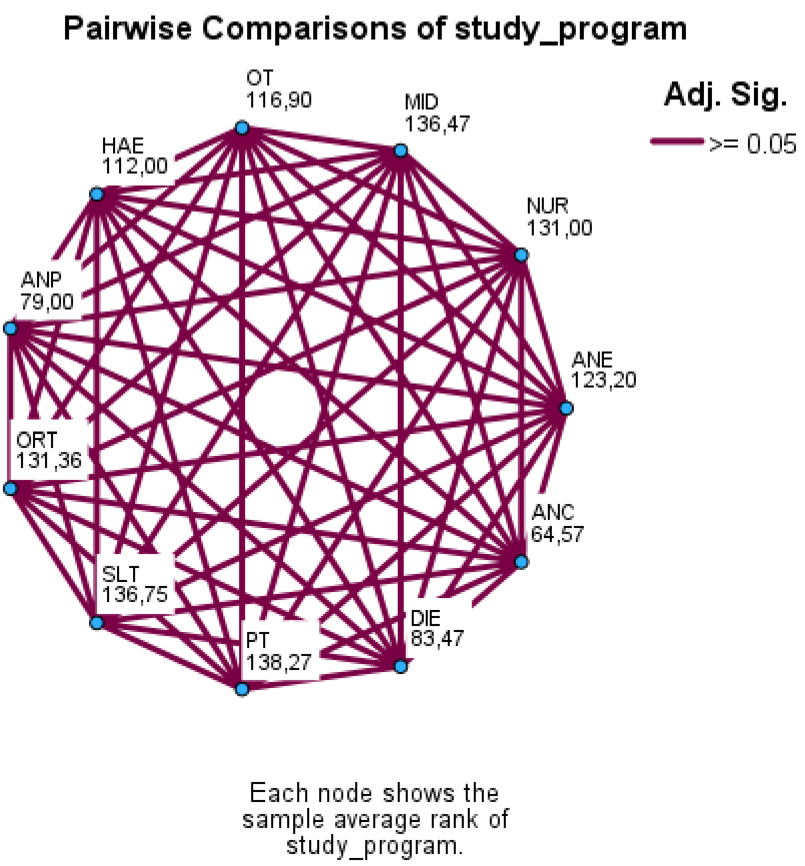

Figure S1: Pairwise comparisons of telehealth interest by study program

Table S1: Pairwise comparisons of telehealth interest by study program

Sample 1-Sample 2	Test Statistic	Std. Error	Std. Test Statistic	Sig.	Adj. Sig.[a]
ANC vs. ANP	14.429	30.506	.473	.636	1.000
ANC vs. DIE	18.902	28.360	.667	.505	1.000
ANC vs. HAE	47.429	28.572	1.660	.097	1.000
ANC vs. OT	52.329	28.169	1.858	.063	1.000
ANC vs. ANE	58.629	29.361	1.997	.046	1.000
ANC vs. NUR	66.429	27.105	2.451	.014	.784
ANC vs. ORT	66.792	27.835	2.400	.016	.903
ANC vs. MID	71.899	26.623	2.701	.007	.381
ANC vs. SLT	72.179	27.553	2.620	.009	.484
ANC vs. PT	73.695	26.061	2.828	.005	.258
ANP vs. DIE	4.474	23.652	.189	.850	1.000
ANP vs. HAE	33.000	23.905	1.380	.167	1.000
ANP vs. OT	37.900	23.422	1.618	.106	1.000
ANP vs. ANE	44.200	24.843	1.779	.075	1.000
ANP vs. NUR	52.000	22.131	2.350	.019	1.000
ANP vs. ORT	52.364	23.019	2.275	.023	1.000
ANP vs. MID	57.471	21.538	2.668	.008	.419
ANP vs. SLT	57.750	22.678	2.547	.011	.598
ANP vs. PT	59.267	20.840	2.844	.004	.245
DIE vs. HAE	28.526	21.098	1.352	.176	1.000
DIE vs. OT	33.426	20.549	1.627	.104	1.000
DIE vs. ANE	-39.726	22.155	-1.793	.073	1.000
DIE vs. NUR	-47.526	19.065	-2.493	.013	.697
DIE vs. ORT	-47.890	20.089	-2.384	.017	.942
DIE vs. MID	52.997	18.373	2.885	.004	.216
DIE vs. SLT	53.276	19.697	2.705	.007	.376
DIE vs. PT	54.793	17.549	3.122	.002	.099
HAE vs. OT	-4.900	20.840	-.235	.814	1.000
HAE vs. ANE	-11.200	22.425	-.499	.617	1.000
HAE vs. NUR	-19.000	19.378	-.980	.327	1.000
HAE vs. ORT	-19.364	20.386	-.950	.342	1.000
HAE vs. MID	-24.471	18.697	-1.309	.191	1.000
HAE vs. SLT	24.750	20.000	1.237	.216	1.000
HAE vs. PT	26.267	17.889	1.468	.142	1.000
OT vs. ANE	-6.300	21.909	-.288	.774	1.000
OT vs. NUR	-14.100	18.779	-.751	.453	1.000
OT vs. ORT	-14.464	19.817	-.730	.465	1.000
OT vs. MID	-19.571	18.076	-1.083	.279	1.000

OT vs. SLT	19.850	19.420	1.022	.307	1.000
OT vs. PT	21.367	17.238	1.240	.215	1.000
ANE vs. NUR	7.800	20.524	.380	.704	1.000
ANE vs. ORT	8.164	21.478	.380	.704	1.000
ANE vs. MID	13.271	19.882	.667	.504	1.000
ANE vs. SLT	13.550	21.112	.642	.521	1.000
ANE vs. PT	15.067	19.124	.788	.431	1.000
NUR vs. ORT	.364	18.274	.020	.984	1.000
NUR vs. MID	5.471	16.369	.334	.738	1.000
NUR vs. SLT	5.750	17.843	.322	.747	1.000
NUR vs. PT	7.267	15.439	.471	.638	1.000
ORT vs. MID	5.107	17.551	.291	.771	1.000
ORT vs. SLT	5.386	18.933	.285	.776	1.000
ORT vs. PT	6.903	16.687	.414	.679	1.000
MID vs. SLT	.279	17.101	.016	.987	1.000
MID vs. PT	1.796	14.575	.123	.902	1.000
SLT vs. PT	1.517	16.213	.094	.925	1.000

Each row tests the null hypothesis that the Sample 1 and Sample 2 distributions are the same.

Asymptotic significances (2-sided tests) are displayed. The significance level is 0.05.

a. Significance values have been adjusted by the Bonferroni correction for multiple tests.

Study program abbreviations: ANC … Advanced Nursing Counseling MSc, ANE … Advanced Nursing Education MSc, ANP … Advanced Nursing Practice MSc, DIE … Dietetics BSc, HAE … Health Assisting Engineering MSc, MID … Midwifery MSc, NUR … Health Care and Nursing BSc, ORT … Orthoptics BSc, OT … Occupational Therapy BSc, PT … Physiotherapy BSc, SLT … Speech & Language Therapy BSc

Hypothesis Test Summary by Study Program

	Null Hypothesis	Test	Sig.[a,b]	Decision
1	The distribution of telehealth interest is the same across categories of study program.	Independent-Samples Kruskal-Wallis Test	0.005	Reject the null hypothesis.
2	The distribution of **telehealth knowledge** is the same across categories of **study program**.	Independent-Samples Kruskal-Wallis Test	<0.001	Reject the null hypothesis.
3	The distribution of importance of telehealth education is the same across categories of study program.	Independent-Samples Kruskal-Wallis Test	<0.001	Reject the null hypothesis.
4	The distribution of role after pandemic is the same across categories of study program.	Independent-Samples Kruskal-Wallis Test	0.004	Reject the null hypothesis.

a. The significance level is 0.05.

b. Asymptotic significance is displayed.

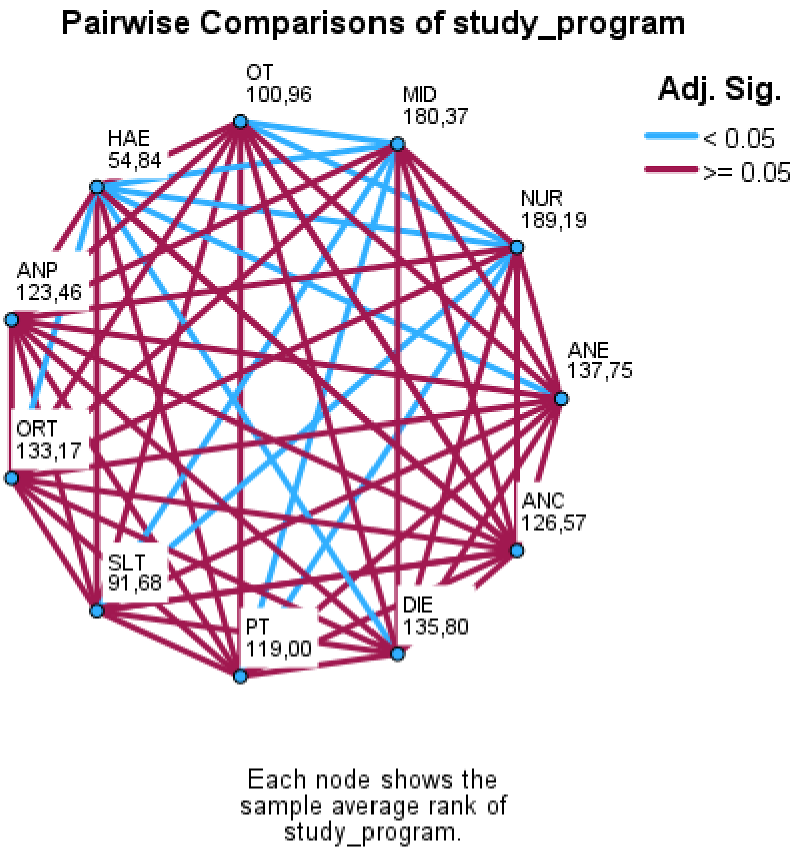

Figure S2: Pairwise comparisons of telehealth knowledge by study program

Table S2: Pairwise comparisons of telehealth knowledge by study program

Sample 1-Sample 2	Test Statistic	Std. Error	Std. Test Statistic	Sig.	Adj. Sig.[a]
HAE vs. SLT	36.838	22.132	1.664	.096	1.000
HAE vs. OT	-46.116	22.330	-2.065	.039	1.000
HAE vs. PT	64.158	19.769	3.245	.001	.065
HAE vs. ANP	-68.619	26.174	-2.622	.009	.481
HAE vs. ANC	-71.729	32.152	-2.231	.026	1.000
**HAE vs. ORT**	-78.332	22.544	-3.475	<.001	**.028**
**HAE vs. DIE**	-80.958	23.296	-3.475	<.001	**.028**
**HAE vs. ANE**	-82.908	24.674	-3.360	<.001	**.043**
**HAE vs. MID**	-125.529	20.722	-6.058	<.001	**.000**
**HAE vs. NUR**	-134.345	21.061	-6.379	<.001	**.000**
SLT vs. OT	-9.278	20.781	-.446	.655	1.000
SLT vs. PT	27.320	18.001	1.518	.129	1.000
SLT vs. ANP	-31.782	24.865	-1.278	.201	1.000
SLT vs. ANC	-34.891	31.096	-1.122	.262	1.000
SLT vs. ORT	-41.494	21.010	-1.975	.048	1.000
SLT vs. DIE	-44.120	21.816	-2.022	.043	1.000
SLT vs. ANE	-46.070	23.281	-1.979	.048	1.000
**SLT vs. MID**	-88.691	19.042	-4.658	<.001	**.000**
**SLT vs. NUR**	-97.507	19.411	-5.023	<.001	**.000**
OT vs. PT	18.042	18.244	.989	.323	1.000
OT vs. ANP	-22.503	25.042	-.899	.369	1.000
OT vs. ANC	-25.613	31.237	-.820	.412	1.000
OT vs. ORT	-32.216	21.219	-1.518	.129	1.000
OT vs. DIE	-34.842	22.017	-1.583	.114	1.000
OT vs. ANE	-36.792	23.470	-1.568	.117	1.000
**OT vs. MID**	-79.413	19.272	-4.121	<.001	**.002**
**OT vs. NUR**	-88.229	19.636	-4.493	<.001	**.000**
PT vs. ANP	-4.462	22.788	-.196	.845	1.000
PT vs. ANC	-7.571	29.461	-.257	.797	1.000
PT vs. ORT	-14.174	18.505	-.766	.444	1.000
PT vs. DIE	-16.800	19.414	-.865	.387	1.000
PT vs. ANE	-18.750	21.048	-.891	.373	1.000
**PT vs. MID**	-61.371	16.236	-3.780	<.001	**.009**
**PT vs. NUR**	-70.187	16.666	-4.211	<.001	**.001**
ANP vs. ANC	-3.110	34.091	-.091	.927	1.000
ANP vs. ORT	9.712	25.233	.385	.700	1.000
ANP vs. DIE	12.338	25.907	.476	.634	1.000
ANP vs. ANE	14.288	27.153	.526	.599	1.000

ANP vs. MID	56.910	23.619	2.410	.016	.879
ANP vs. NUR	65.726	23.917	2.748	.006	.330
ANC vs. ORT	6.602	31.390	.210	.833	1.000
ANC vs. DIE	9.229	31.935	.289	.773	1.000
ANC vs. ANE	11.179	32.953	.339	.734	1.000
ANC vs. MID	53.800	30.108	1.787	.074	1.000
ANC vs. NUR	62.616	30.343	2.064	.039	1.000
ORT vs. DIE	2.626	22.233	.118	.906	1.000
ORT vs. ANE	-4.576	23.673	-.193	.847	1.000
ORT vs. MID	47.198	19.519	2.418	.016	.858
ORT vs. NUR	-56.014	19.879	-2.818	.005	.266
DIE vs. ANE	-1.950	24.391	-.080	.936	1.000
DIE vs. MID	44.571	20.383	2.187	.029	1.000
DIE vs. NUR	-53.387	20.728	-2.576	.010	.550
ANE vs. MID	42.621	21.945	1.942	.052	1.000
ANE vs. NUR	51.438	22.265	2.310	.021	1.000
MID vs. NUR	-8.816	17.786	-.496	.620	1.000

Each row tests the null hypothesis that the Sample 1 and Sample 2 distributions are the same.

Asymptotic significances (2-sided tests) are displayed. The significance level is 0.05.

a. Significance values have been adjusted by the Bonferroni correction for multiple tests.

Study program abbreviations: ANC … Advanced Nursing Counseling MSc, ANE … Advanced Nursing Education MSc, ANP … Advanced Nursing Practice MSc, DIE … Dietetics BSc, HAE … Health Assisting Engineering MSc, MID … Midwifery MSc, NUR … Health Care and Nursing BSc, ORT … Orthoptics BSc, OT … Occupational Therapy BSc, PT … Physiotherapy BSc, SLT … Speech & Language Therapy BSc

Hypothesis Test Summary by Study Program

	Null Hypothesis	Test	Sig.[a,b]	Decision
1	The distribution of telehealth interest is the same across categories of study program.	Independent-Samples Kruskal-Wallis Test	0.005	Reject the null hypothesis.
2	The distribution of telehealth knowledge is the same across categories of study program.	Independent-Samples Kruskal-Wallis Test	<0.001	Reject the null hypothesis.
3	The distribution of **importance of telehealth education** is the same across categories of **study program**.	Independent-Samples Kruskal-Wallis Test	<0.001	Reject the null hypothesis.
4	The distribution of role after pandemic is the same across categories of study program.	Independent-Samples Kruskal-Wallis Test	0.004	Reject the null hypothesis.

a. The significance level is 0.05.

b. Asymptotic significance is displayed.

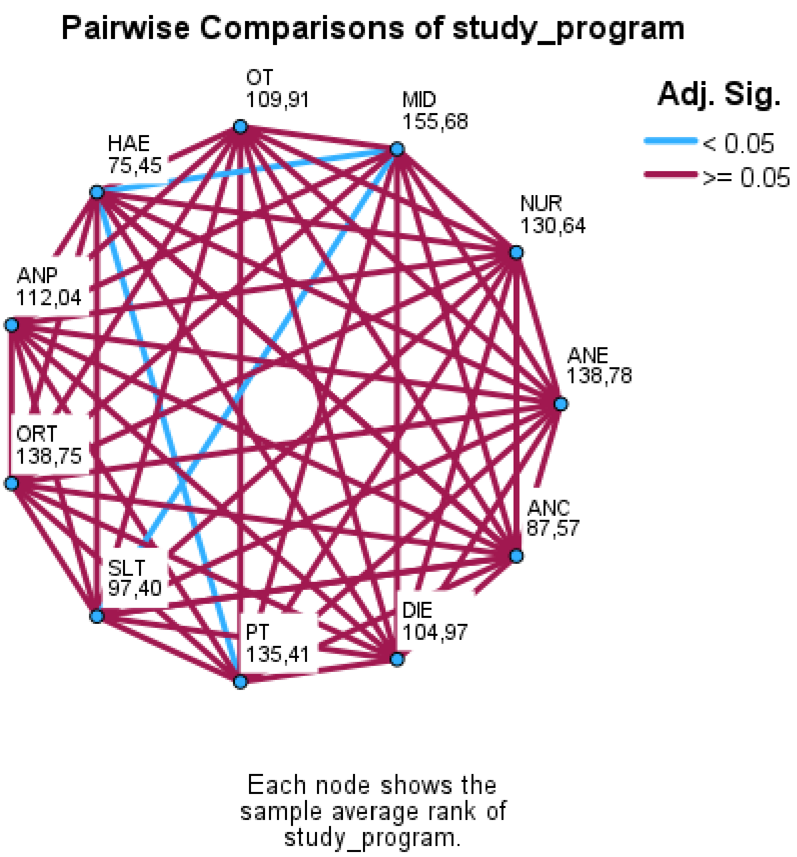

Figure S3: Pairwise comparisons of importance of telehealth education by study program

Table S3: Pairwise comparisons of importance of telehealth education by study program

Sample 1-Sample 2	Test Statistic	Std. Error	Std. Test Statistic	Sig.	Adj. Sig.[a]
HAE vs. ANC	-12.124	27.893	-.435	.664	1.000
HAE vs. SLT	21.948	19.372	1.133	.257	1.000
HAE vs. DIE	-29.526	20.468	-1.443	.149	1.000
HAE vs. OT	-34.462	19.758	-1.744	.081	1.000
HAE vs. ANP	-36.594	23.262	-1.573	.116	1.000
HAE vs. NUR	-55.195	18.751	-2.944	.003	.178
**HAE vs. PT**	59.962	17.318	3.462	<.001	**.029**
HAE vs. ORT	-63.303	20.210	-3.132	.002	.095
HAE vs. ANE	-63.334	21.406	-2.959	.003	.170
**HAE vs. MID**	-80.234	18.168	-4.416	<.001	**.001**
ANC vs. SLT	9.824	27.099	.363	.717	1.000
ANC vs. DIE	17.402	27.893	.624	.533	1.000
ANC vs. OT	22.338	27.376	.816	.415	1.000
ANC vs. ANP	24.470	30.003	.816	.415	1.000
ANC vs. NUR	43.071	26.659	1.616	.106	1.000
ANC vs. PT	47.838	25.671	1.863	.062	1.000
ANC vs. ORT	51.179	27.704	1.847	.065	1.000
ANC vs. ANE	51.210	28.588	1.791	.073	1.000
ANC vs. MID	68.110	26.252	2.595	.009	.521
SLT vs. DIE	-7.578	19.372	-.391	.696	1.000
SLT vs. OT	-12.513	18.621	-.672	.502	1.000
SLT vs. ANP	-14.646	22.304	-.657	.511	1.000
SLT vs. NUR	-33.247	17.549	-1.895	.058	1.000
SLT vs. PT	38.013	16.009	2.375	.018	.966
SLT vs. ORT	-41.354	19.100	-2.165	.030	1.000
SLT vs. ANE	-41.385	20.361	-2.033	.042	1.000
**SLT vs. MID**	-58.286	16.924	-3.444	<.001	**.032**
DIE vs. OT	4.935	19.758	.250	.803	1.000
DIE vs. ANP	-7.068	23.262	-.304	.761	1.000
DIE vs. NUR	-25.669	18.751	-1.369	.171	1.000
DIE vs. PT	30.435	17.318	1.757	.079	1.000
DIE vs. ORT	-33.776	20.210	-1.671	.095	1.000
DIE vs. ANE	-33.808	21.406	-1.579	.114	1.000
DIE vs. MID	50.708	18.168	2.791	.005	.289
OT vs. ANP	-2.133	22.640	-.094	.925	1.000
OT vs. NUR	-20.734	17.973	-1.154	.249	1.000
OT vs. PT	25.500	16.473	1.548	.122	1.000

OT vs. ORT	-28.841	19.491	-1.480	.139	1.000
OT vs. ANE	-28.872	20.728	-1.393	.164	1.000
OT vs. MID	-45.773	17.364	-2.636	.008	.461
ANP vs. NUR	18.601	21.767	.855	.393	1.000
ANP vs. PT	23.367	20.545	1.137	.255	1.000
ANP vs. ORT	26.708	23.036	1.159	.246	1.000
ANP vs. ANE	26.740	24.091	1.110	.267	1.000
ANP vs. MID	43.640	21.266	2.052	.040	1.000
NUR vs. PT	4.766	15.251	.313	.755	1.000
NUR vs. ORT	8.107	18.470	.439	.661	1.000
NUR vs. ANE	-8.138	19.771	-.412	.681	1.000
NUR vs. MID	25.039	16.209	1.545	.122	1.000
PT vs. ORT	-3.341	17.013	-.196	.844	1.000
PT vs. ANE	-3.372	18.417	-.183	.855	1.000
PT vs. MID	-20.273	14.528	-1.395	.163	1.000
ORT vs. ANE	-.031	21.160	-.001	.999	1.000
ORT vs. MID	16.932	17.877	.947	.344	1.000
ANE vs. MID	16.901	19.218	.879	.379	1.000

Each row tests the null hypothesis that the Sample 1 and Sample 2 distributions are the same.

Asymptotic significances (2-sided tests) are displayed. The significance level is 0.05.

a. Significance values have been adjusted by the Bonferroni correction for multiple tests.

Study program abbreviations: ANC … Advanced Nursing Counseling MSc, ANE … Advanced Nursing Education MSc, ANP … Advanced Nursing Practice MSc, DIE … Dietetics BSc, HAE … Health Assisting Engineering MSc, MID … Midwifery MSc, NUR … Health Care and Nursing BSc, ORT … Orthoptics BSc, OT … Occupational Therapy BSc, PT … Physiotherapy BSc, SLT … Speech & Language Therapy BSc

Hypothesis Test Summary by Study Program

	Null Hypothesis	Test	Sig.[a,b]	Decision
1	The distribution of telehealth interest is the same across categories of study program.	Independent-Samples Kruskal-Wallis Test	0.005	Reject the null hypothesis.
2	The distribution of telehealth knowledge is the same across categories of study program.	Independent-Samples Kruskal-Wallis Test	<0.001	Reject the null hypothesis.
3	The distribution of importance of telehealth education is the same across categories of study program.	Independent-Samples Kruskal-Wallis Test	<0.001	Reject the null hypothesis.
4	The distribution of **role after pandemic** is the same across categories of **study program**.	Independent-Samples Kruskal-Wallis Test	0.004	Reject the null hypothesis.

a. The significance level is 0.05.

b. Asymptotic significance is displayed.

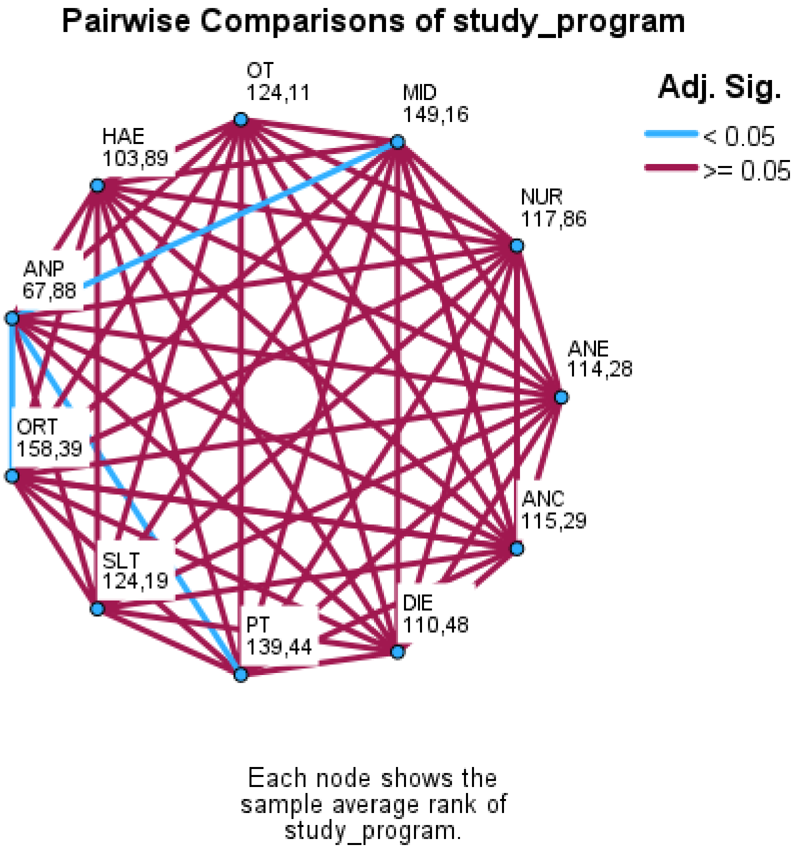

Figure S4: Pairwise comparisons of role after the pandemic by study program

Table S4: Pairwise comparisons of role after the pandemic by study program

Sample 1-Sample 2	Test Statistic	Std. Error	Std. Test Statistic	Sig.	Adj. Sig.[a]
ANP vs. HAE	36.020	24.012	1.500	.134	1.000
ANP vs. DIE	42.600	23.778	1.792	.073	1.000
ANP vs. ANE	46.406	24.868	1.866	.062	1.000
ANP vs. ANC	-47.411	30.971	-1.531	.126	1.000
ANP vs. NUR	49.982	22.468	2.225	.026	1.000
ANP vs. OT	56.234	23.190	2.425	.015	.842
ANP vs. SLT	56.313	23.023	2.446	.014	.795
**ANP vs. PT**	71.561	21.062	3.398	<.001	**.037**
**ANP vs. MID**	81.287	21.866	3.718	<.001	**.011**
**ANP vs. ORT**	90.511	23.370	3.873	<.001	**.006**
HAE vs. DIE	-6.580	20.862	-.315	.752	1.000
HAE vs. ANE	-10.387	22.096	-.470	.638	1.000
HAE vs. ANC	-11.391	28.792	-.396	.692	1.000
HAE vs. NUR	-13.962	19.356	-.721	.471	1.000
HAE vs. OT	-20.214	20.188	-1.001	.317	1.000
HAE vs. SLT	20.293	19.997	1.015	.310	1.000
HAE vs. PT	35.541	17.703	2.008	.045	1.000
HAE vs. MID	-45.267	18.652	-2.427	.015	.838
HAE vs. ORT	-54.492	20.395	-2.672	.008	.415
DIE vs. ANE	-3.806	21.842	-.174	.862	1.000
DIE vs. ANC	-4.811	28.598	-.168	.866	1.000
DIE vs. NUR	-7.382	19.065	-.387	.699	1.000
DIE vs. OT	13.634	19.910	.685	.493	1.000
DIE vs. SLT	13.713	19.716	.696	.487	1.000
DIE vs. PT	28.961	17.385	1.666	.096	1.000
DIE vs. MID	38.687	18.351	2.108	.035	1.000
DIE vs. ORT	-47.911	20.119	-2.381	.017	.949
ANE vs. ANC	-1.004	29.510	-.034	.973	1.000
ANE vs. NUR	3.576	20.408	.175	.861	1.000
ANE vs. OT	9.827	21.199	.464	.643	1.000
ANE vs. SLT	9.906	21.017	.471	.637	1.000
ANE vs. PT	25.155	18.848	1.335	.182	1.000
ANE vs. MID	34.881	19.742	1.767	.077	1.000
ANE vs. ORT	44.105	21.396	2.061	.039	1.000
ANC vs. NUR	2.571	27.518	.093	.926	1.000
ANC vs. OT	8.823	28.110	.314	.754	1.000
ANC vs. SLT	8.902	27.973	.318	.750	1.000
ANC vs. PT	24.150	26.382	.915	.360	1.000

ANC vs. MID	33.876	27.028	1.253	.210	1.000
ANC vs. ORT	43.101	28.259	1.525	.127	1.000
NUR vs. OT	6.252	18.325	.341	.733	1.000
NUR vs. SLT	6.330	18.115	.349	.727	1.000
NUR vs. PT	21.579	15.546	1.388	.165	1.000
NUR vs. MID	31.305	16.618	1.884	.060	1.000
NUR vs. ORT	40.529	18.553	2.185	.029	1.000
OT vs. SLT	.079	19.002	.004	.997	1.000
OT vs. PT	15.327	16.571	.925	.355	1.000
OT vs. MID	-25.053	17.581	-1.425	.154	1.000
OT vs. ORT	-34.278	19.420	-1.765	.078	1.000
SLT vs. PT	15.249	16.338	.933	.351	1.000
SLT vs. MID	-24.974	17.361	-1.439	.150	1.000
SLT vs. ORT	-34.199	19.221	-1.779	.075	1.000
PT vs. MID	-9.726	14.661	-.663	.507	1.000
PT vs. ORT	-18.950	16.822	-1.127	.260	1.000
MID vs. ORT	-9.225	17.818	-.518	.605	1.000

Each row tests the null hypothesis that the Sample 1 and Sample 2 distributions are the same.

Asymptotic significances (2-sided tests) are displayed. The significance level is 0.05.

a. Significance values have been adjusted by the Bonferroni correction for multiple tests.

Study program abbreviations: ANC … Advanced Nursing Counseling MSc, ANE … Advanced Nursing Education MSc, ANP … Advanced Nursing Practice MSc, DIE … Dietetics BSc, HAE … Health Assisting Engineering MSc, MID … Midwifery MSc, NUR … Health Care and Nursing BSc, ORT … Orthoptics BSc, OT … Occupational Therapy BSc, PT … Physiotherapy BSc, SLT … Speech & Language Therapy BSc

Hypothesis Test Summary by Gender

	Null Hypothesis	Test	Sig.[a,b]	Decision
1	The distribution of telehealth interest is the same across categories of gender.	Independent-Samples Kruskal-Wallis Test	0.628	Retain the null hypothesis.
2	The distribution of telehealth knowledge is the same across categories of gender.	Independent-Samples Kruskal-Wallis Test	0.190	Retain the null hypothesis.
3	The distribution of importance of telehealth education is the same across categories of gender.	Independent-Samples Kruskal-Wallis Test	0.727	Retain the null hypothesis.
4	The distribution of role after pandemic is the same across categories of gender.	Independent-Samples Kruskal-Wallis Test	0.551	Retain the null hypothesis.

a. The significance level is 0.05.

b. Asymptotic significance is displayed.

Hypothesis Test Summary by Generation

	Null Hypothesis	Test	Sig.[a,b]	Decision
1	The distribution of telehealth interest is the same across categories of generation.	Independent-Samples Kruskal-Wallis Test	0.136	Retain the null hypothesis.
2	The distribution of telehealth knowledge is the same across categories of generation.	Independent-Samples Kruskal-Wallis Test	0.194	Retain the null hypothesis.
3	The distribution of **importance of telehealth education** is the same across categories of **generation**.	Independent-Samples Kruskal-Wallis Test	0.012	Reject the null hypothesis.
4	The distribution of role after pandemic is the same across categories of generation.	Independent-Samples Kruskal-Wallis Test	0.064	Retain the null hypothesis.

a. The significance level is 0.05.

b. Asymptotic significance is displayed.

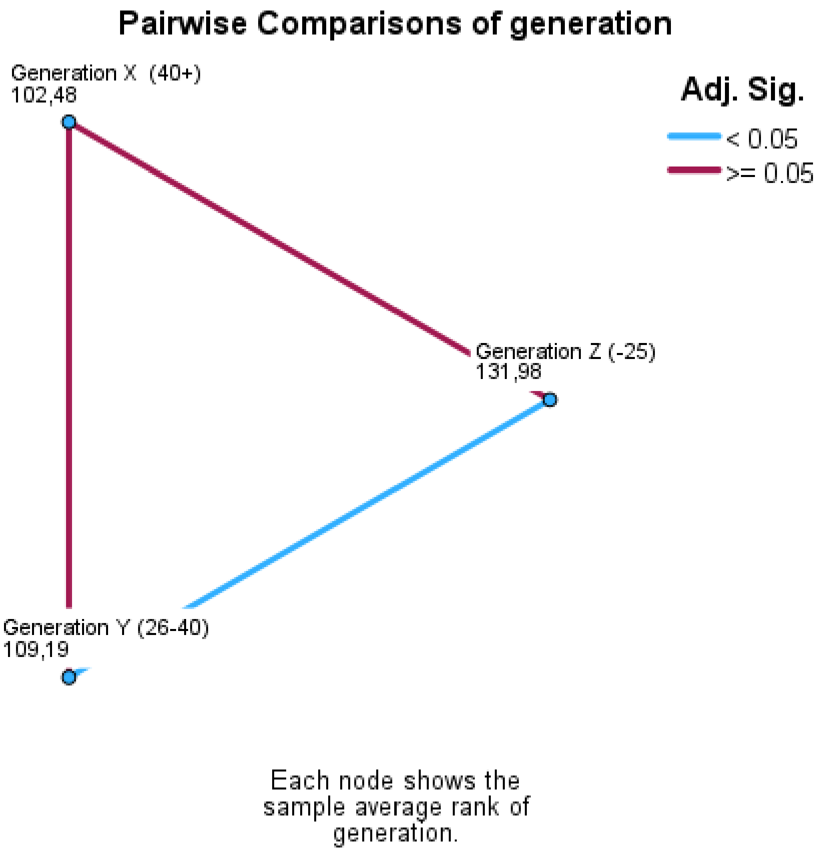

Figure S5: Pairwise comparisons of role after the pandemic by generation

Table S5: Pairwise comparisons of role after the pandemic by generation

Sample 1-Sample 2	Test Statistic	Std. Error	Std. Test Statistic	Sig.	Adj. Sig.[a]
Generation X (40+) vs. Generation Y (26-40)	6.715	15.343	.438	.662	1.000
Generation X (40+) vs. Generation Z (-25)	29.499	14.409	2.047	.041	.122
**Generation Y (26-40) vs. Generation Z (-25)**	22.785	9.013	2.528	.011	**.034**

Each row tests the null hypothesis that the Sample 1 and Sample 2 distributions are the same.

Asymptotic significances (2-sided tests) are displayed. The significance level is 0.05.

a. Significance values have been adjusted by the Bonferroni correction for multiple tests.

Hypothesis Test Summary by Semester

	Null Hypothesis	Test	Sig.[a,b]	Decision
1	The distribution of telehealth interest is the same across categories of semester.	Independent-Samples Kruskal-Wallis Test	0.086	Retain the null hypothesis.
2	The distribution of telehealth knowledge is the same across categories of semester.	Independent-Samples Kruskal-Wallis Test	<0.001	Reject the null hypothesis.
3	The distribution of importance of telehealth education is the same across categories of semester.	Independent-Samples Kruskal-Wallis Test	0.091	Retain the null hypothesis.
4	The distribution of **role after pandemic** is the same across categories of **semester**.	Independent-Samples Kruskal-Wallis Test	0.008	Reject the null hypothesis.

a. The significance level is 0.05.

b. Asymptotic significance is displayed.

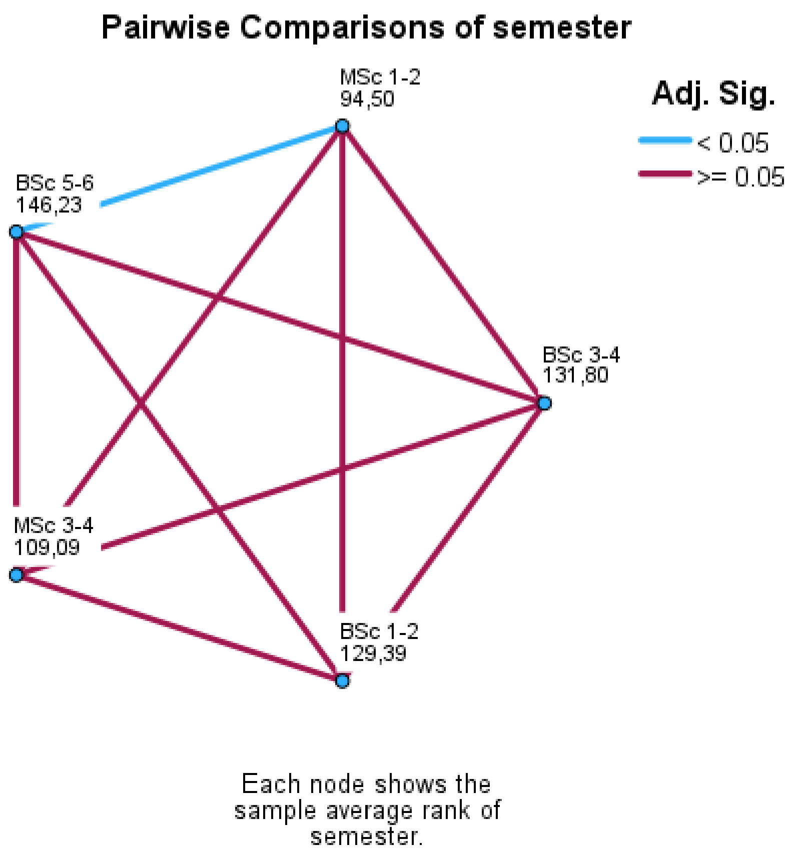

Figure S6: Pairwise comparisons of role after pandemic by semester

Table S6: Pairwise comparisons of role after pandemic by semester

Sample 1-Sample 2	Test Statistic	Std. Error	Std. Test Statistic	Sig.	Adj. Sig.[a]
MSc 1-2 vs. MSc 3-4	-14.591	18.035	-.809	.419	1.000
MSc 1-2 vs. BSc 1-2	34.887	13.275	2.628	.009	.086
MSc 1-2 vs. BSc 3-4	37.300	14.255	2.617	.009	.089
**MSc 1-2 vs. BSc 5-6**	51.732	15.361	3.368	<.001	**.008**
MSc 3-4 vs. BSc 1-2	20.296	15.378	1.320	.187	1.000
MSc 3-4 vs. BSc 3-4	22.709	16.231	1.399	.162	1.000
MSc 3-4 vs. BSc 5-6	37.141	17.210	2.158	.031	.309
BSc 1-2 vs. BSc 3-4	-2.413	10.695	-.226	.821	1.000
BSc 1-2 vs. BSc 5-6	-16.845	12.130	-1.389	.165	1.000
BSc 3-4 vs. BSc 5-6	-14.432	13.195	-1.094	.274	1.000

Each row tests the null hypothesis that the Sample 1 and Sample 2 distributions are the same.
Asymptotic significances (2-sided tests) are displayed. The significance level is 0.05.
a. Significance values have been adjusted by the Bonferroni correction for multiple tests.
